# Electrochemical Activation of Li_2_MnO_3_ Electrodes at 0 °C and Its Impact on the Subsequent Performance at Higher Temperatures

**DOI:** 10.3390/ma13194388

**Published:** 2020-10-01

**Authors:** Francis Amalraj Susai, Michael Talianker, Jing Liu, Rosy, Tanmoy Paul, Yehudit Grinblat, Evan Erickson, Malachi Noked, Larisa Burstein, Anatoly I. Frenkel, Yoed Tsur, Boris Markovsky, Doron Aurbach

**Affiliations:** 1 Department of Chemistry and Institute of Nanotechnology and Advanced Materials, Bar-Ilan University, Ramat-Gan 52900, Israel; sfamalraj@gmail.com (F.A.S.); rrosysharma@gmail.com (R.S.); Judith.Grinblat@biu.ac.il (Y.G.); evan.m.erickson@gmail.com (E.E.); malachi.noked@biu.ac.il (M.N.); 2 Department of Materials Engineering, Ben-Gurion University of the Negev, Beer-Sheva 84105, Israel; mtalianker973@gmail.com; 3 Department of Physics, Manhattan College, Riverdale, New York, NY 10471, USA; jing.liu@huskers.unl.edu; 4 Department of Chemical Engineering and the Grand Technion Energy Program, Technion-Israel Institute of Technology, Haifa 3200003, Israel; tanmoykent@gmail.com (T.P.); tsur@technion.ac.il (Y.T.); 5 The Wolfson Applied Materials Research Centre, Tel-Aviv University, Tel-Aviv 69978, Israel; bursten@tauex.tau.ac.il; 6 Department of Materials Science and Chemical Engineering, Stony Brook University, Stony Brook, New York, NY 11794, USA; frenkel@bnl.gov

**Keywords:** lithium-ion batteries, Li- and Mn-rich materials, Li_2_MnO_3_ activation at 0 °C, stabilized cycling, decreased the voltage hysteresis, layered-to-spinel transition, bulk and surface characteristics

## Abstract

This work continues our systematic study of Li- and Mn- rich cathodes for lithium-ion batteries. We chose Li_2_MnO_3_ as a model electrode material with the aim of correlating the improved electrochemical characteristics of these cathodes initially activated at 0 °C with the structural evolution of Li_2_MnO_3_, oxygen loss, formation of per-oxo like species (O_2_^2−^) and the surface chemistry. It was established that performing a few initial charge/discharge (activation) cycles of Li_2_MnO_3_ at 0 °C resulted in increased discharge capacity and higher capacity retention, and decreased and substantially stabilized the voltage hysteresis upon subsequent cycling at 30 °C or at 45 °C. In contrast to the activation of Li_2_MnO_3_ at these higher temperatures, Li_2_MnO_3_ underwent step-by-step activation at 0 °C, providing a stepwise traversing of the voltage plateau at >4.5 V during initial cycling. Importantly, these findings agree well with our previous studies on the activation at 0 °C of 0.35Li_2_MnO_3_·0.65Li[Mn_0.45_Ni_0.35_Co_0.20_]O_2_ materials. The stability of the interface developed at 0 °C can be ascribed to the reduced interactions of the per-oxo-like species formed and the oxygen released from Li_2_MnO_3_ with solvents in ethylene carbonate–methyl-ethyl carbonate/LiPF_6_ solutions. Our TEM studies revealed that typically, upon initial cycling both at 0 °C and 30 °C, Li_2_MnO_3_ underwent partial structural layered-to-spinel (Li_2_Mn_2_O_4_) transition.

## 1. Introduction

Among various materials for cathodes for advanced Li-ion batteries (LIB), Li- and Mn- rich oxides like Li_1+x_Ni_y_Co_z_Mn_0.5+w_O_2_ (x + y + z + w = 0.5) or xLi_2_MnO_3_·(1−x)LiMO_2_ (M = Mn, Ni, Co) are the most promising because of their high discharge capacity (>250 mAh/g) after the first activation charge–discharge cycle and high energy density, i.e., ~1000 Wh/kg. These materials have attracted much attention since they were first synthesized and introduced by Thackeray et al. [1,2,3,4,5,6,7]. It should be emphasized that during the last decade, sodium and potassium ion batteries—in which the energy storage mechanism is similar to that in LIBs—have been proposed and studied worldwide [8,9,10,11,12,13,14]. Electrodes comprising high-energy lithiated Ni, Co, Mn oxides (HE-NCM) suffer from fast capacity fading, large voltage hysteresis, and transition metal dissolution during prolonged cycling [5,15,16,17,18,19,20,21,22,23,24,25]. Therefore, researchers have proposed several approaches to stabilize their electrochemical performance, such as surface coatings by oxides and salts, for instance Al_2_O_3_, TiO_2_, ZrO_2_, AlF_3_ [26,27,28], cationic or anionic lattice doping with Na^+^, Mg^2+^, Al^3+^, F^−^, etc. [29,30,31,32,33], and gas treatment at high tempature with NH_3_, SO_2_, CO_2_, or F_2_ [34,35,36,37]. Additionally, Thackeray et al. [38,39] showed that treatment of HE-NCM materials with inorganic acids (H_2_SO_4_, HNO_3_) resulted in stable electrochemical behavior. We recently demonstrated [40] that stable cycling and higher rate capability and lower voltage hysteresis of HE-NCM cathodes in Li-cells can be achieved after the treatment of these materials with trimesic or terephthalic acids at 600 °C. An important finding was reported in our previous work [41], i.e., that performing initial (activation) cycling of HE-NCM electrodes at low temperature, for instance at 0 °C or at 15 °C, resulted in higher discharge capacity and decreased and stabilized voltage hysteresis compared to electrodes initially activated and cycled at the higher temperatures (e.g., 30° and 45 °C). In that work, we used 0.35Li_2_MnO_3_·0.65Li[Ni_0.35_Mn_0.45_Co_0.20_]O_2_, a material reffered as a two-phase system consisting of layered, structurally compatible components: Li_2_MnO_3_ (monoclinic, space group *C*2/*m*) and Li[Ni_0.35_Mn_0.45_Co_0.20_]O_2_ (rhombohedral, space group *R-3m*) integrated on a nanoscale [15,18,42,43,44] and demonstrating common *d*-spacing [45]. Li_2_MnO_3_ is attracting attention not only as one of the end-members of the HE-NCM materials, but as a high-capacity cathode with a theoretical capacity of ~460 mAh/g for total Li extraction when charged to potentials >4.6 V. However, Li_2_MnO_3_ can provide high specific capacity (>200 mAh/g) only in electrodes comprising nanosized particles which demonstrate higher capacity and enhanced cycling behavior, compared to those of submicronic or micron-sized materials. Li_2_MnO_3_ plays a critical role in HE-NCM cathodes, providing high capacity during cycling in the potential range of 2.0–4.6 V by introducing a reversible anionic red-ox chemistry, 2O^2−^ → O_2_^2−^+2e^−^ (in the framework of the crystal structure of the oxide). This oxygen redox compensates for almost half of the measured capacity of HE-NCMs in the initial cycles, and more than one-third of the capacity results from reversible oxygen redox after 70 cycles, as established by Tarascon et al. [45]. Despite the numerous experimental [46,47,48,49,50,51,52] and theoretical [53,54,55,56,57] studies on Li- and Mn-rich layered materials as cathodes in Li-cells, much effort is still required to reveal the relationship among the composition of these materials and their structure, layered-to-spinel phase transformations, and enhanced electrochemical characteristics, depending on the cycling conditions (e.g., potential window, current density applied), electrolyte solutions, and temperature.

The main goal of the present work, which is a continuation of our recent research [41], was to study how the initial (activation) cycling of Li_2_MnO_3_ electrodes at low temperature (0 °C) influences their subsequent electrochemical performance at the higher temperatures (for instance, 30 °C and 45 °C). We also aimed to correlate the improved electrochemical characteristics of these electrodes—i.e., in terms of their stabililized capacity and voltage hysteresis—with the structural evolution of Li_2_MnO_3_, oxygen loss, the formation of per-oxo like species (O_2_)^n−^, and a problematic surface chemistry. Thus, we hoped to gain a deep understanding of the basic phenomenon of the temperature-dependent activation (“low-T activation”) of xLi_2_MnO_3_·(1−x)Li[Ni-Mn-Co]O_2_ materials at 0 °C.

## 2. Materials and Methods

### 2.1. Synthesis of Li_2_MnO_3_

**Scheme 1:**
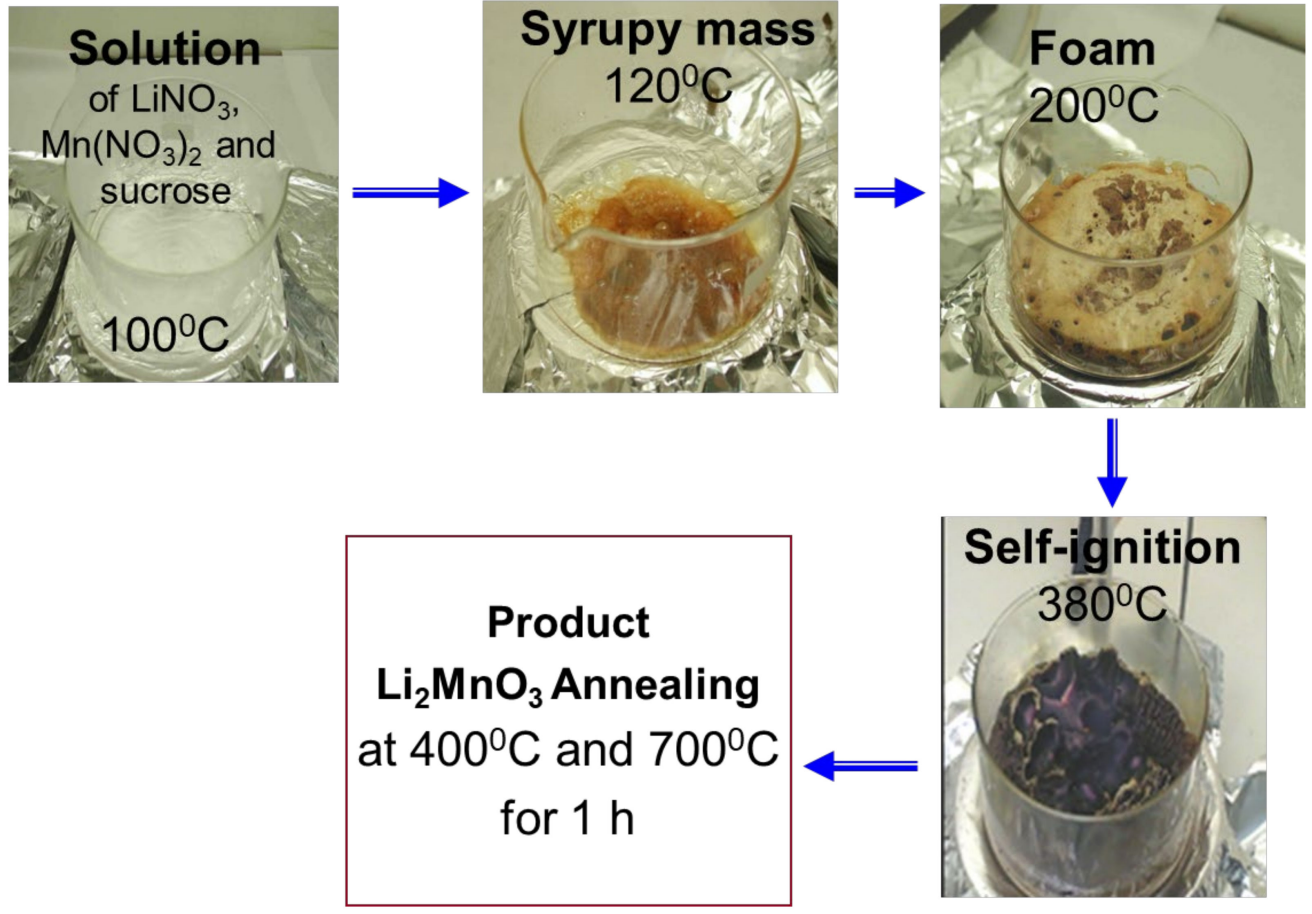
Self-combustion synthesis of Li_2_MnO_3_ from Li- and Mn-nitrates (oxidants) and sucrose (fuel).

Li_2_MnO_3_ was produced by solution-combustion reaction (SCR) using an aqueous solution of lithium nitrate (LiNO_3_) and manganese nitrate (Mn(NO_3_)_2_) (Sigma-Aldrich, St. Louis, MO, USA), which acted as the oxidant, and sucrose C_12_H_22_O_11_ as the fuel, similarly to previous reports [58,59]. The oxidant/fuel ratio was 1:1. Below, we present Scheme 1, which illustrates the process of the SCR. First, by heating the solution of lithium and manganese nitrates and sucrose to 100 °C, the water evaporated slowly and the mixture transformed into a syrupy mass. At ~200 °C, foam was formed and further heating led to the self-ignition of the reactants. The obtained product was amorphous; to obtain a well-crystallized Li_2_MnO_3_, the as-prepared material was ground with pestle and mortar and annealed in an aluminum crucible at 400 and 700 °C for 1 h in air. The individual primary particles obtained were of 30–100 nm in size, some of which were agglomerates of ~1–1.5 µ. A SEM image is presented in Figure S1. The specific active surface area was ~20 m^2^ g^−1^, as measured by the Brunauer, Emmet and Teller (BET) method (Gemini 2375, Micromeritics, multipoint mode). Chemical analysis of the annealed product was carried out using the inductive coupled plasma technique (ICP-AES, spectrometer Ultima-2 from Jobin-Yvon Horiba, Montpellier, France). The composition of the material was Mn:Li = 1:2, corresponding to Li_2_MnO_3_, with a measurement accuracy of ~98%.

### 2.2. Preparation of Electrodes and Electrochemical Cells

Working electrodes (cathodes) for electrochemical cells were prepared by mixing the active material Li_2_MnO_3_ with carbon black super P (from Timcal, Bodio, Switzerland), graphite KS-6 (Timcal, Bodio, Switzerland) and polyvinylidene difluoride binder PVdF (Solef 5130 from Solvay, Brussels, Belgium) at a ratio of 80:10:10 wt.%, in N-methyl pyrrolidone (Sigma-Aldrich, St. Louis, MO, USA) using a Thinky (Tokyo, Japan) planetary vacuum mixer to obtain a slurry. It was then cast onto an aluminum foil (15 µm thick, from Strem chemicals, Newburyport, MA, USA), dried on a hotplate, followed by drying at 120 °C under vacuum overnight.

### 2.3. Electrochemical Measurements

Electrochemical tests were carried out in two-electrode cells of 2325 coin-type configuration (parts from NRC-CNRC, Ottawa, Ontario, Canada). The geometric area of the working electrodes was ~1.5 cm^2^, and the average loading of the active mass was ~3 mg/cm^2^. Counter electrodes were prepared from ~200-μm thick lithium foil (Honjo metal Co.,Osaka, Japan). Electrochemical cells were assembled in a glove box (VAC, from Hawthorne, CA, USA) filled with highly pure argon (5N, Maxima, Ashdod, Israel). The electrolyte solutions (Li battery grade, LP57 from BASF, Ludwigshafen, Germany) comprised 1M LiPF_6_ dissolved in ethyl-methyl carbonate (EMC) and ethylene carbonate (EC), at a weight ratio 7:3. For statistical purposes, we studied the electrochemical perfromance of at least 2–3 cells simultaneously, and the results were averaged. The electrochemical measurements were performed using a multichannel Maccor-2000 battery cycler and a battery test unit model 1470, coupled with a with a frequency response analyser (FRA) model 1255 from Solartron, Inc, Shildon, Durham, UK, driven by Corrware and ZPlot software (from Scribner Associates, Inc., Southern Pines, NC, USA). The alternating voltage amplitude in impedance measurements was 3 mV and the frequency ranged from 100 kHz to 5 mHz. The electrochemical impedance spectroscopy data were analyzed using Impedance Spectroscopy Genetic Programing (ISGP), a matlab based program that finds the distribution function of relaxation times (DFRT) via genetic programming [60,61,62]. ISGP finds an analytical form of the DFRT, which makes it possible to follow the changes in each peak that idealy represent a process. All the potentials in this paper are given vs. Li^+^/Li. Electrochemical tests and online electrochemical mass spectrometry measurements were conducted in thermostats at 0 °C (from Binder, GmbH-Tuttlingen, Germany) and higher temperatures (from MRC, Holon, Israel).

### 2.4. Structural and Surface Studies

X-ray powder diffraction (XRD) (Billerica, MA, USA) measurements were performed in the 2θ range from 10 to 80°, with a step size of 0.02° at 15 sec/step rate. Analysis of the XRD patterns was carried out using the PowderCell program [63] and the Fullprof program, as described elsewhere [64]. Transmission electron microscopy (TEM) studies of the Li_2_MnO_3_ particles were performed with a LaB_6_-200 kV Jeol-2100 electron microscope (Tokyo, Japan), and convergent beam electron diffractions (CBED) were taken using a 7-nm probe size. Samples for the TEM studies were prepared by the methodology described in [65]. Scanning electron microscopy (SEM) images of Li_2_MnO_3_ powder were obtained using E-SEM (environmental scanning electron microscope), Quanta FEG (Thermo Fisher Scientific, Agawam, US). Micro-Raman spectroscopy measurements of electrodes and powders scratched from them were performed at room temperature using a micro-Raman spectrometer from Renishaw inVia (Banbury, UK) equipped with a 514 nm laser, a CCD camera, and an optical Leica microscope. A 50× objective lens to focus the incident beam and an 1800 lines/mm grating were used. For statistical purposes, Raman spectra were recorded from 10–20 locations arbitrarily chosen on a sample. X-ray Photoelectron Spectroscopy (XPS) measurements were performed in ultrahigh vacuum (2.5 × 10^−10^ Torr base pressure) using a 5600 Multi-Technique System (Physical Electronics Inc, MN, USA). The samples were irradiated with an Al Kα monochromated source (1486.6 eV) and the outcome electrons were analyzed by a spherical capacitance analyzer using the slit aperture of 0.8 mm in diameter. High resolution spectra were taken at pass energy of 11.75 eV at increments of 0.05 eV/step to allow precise energy position and peak shape determination. Curve fitting was done with a Gaussian-Lorentzian function using the 5600 Multi-Technique System software (from PHI, Buras, LA, USA). Mn K-edge X-ray absorption fine structure (XAFS) spectroscopic measurements of Li_2_MnO_3_ were carried out at BL2-2 beamline at Stanford Synchrotron Radiation Light source (SSRL) in transmission mode. The Li_2_MnO_3_ sample was ground to a fine powder and brushed on tape for measurements. XAFS data processing and analysis were done using the Athena and Artemis software within the IFEFFIT package [66]. The normalized *k^2^*-weighted EXAFS data were Fourier transformed in a *k* range of 2–11 Å^−1^ for Mn. In the fits, the contributions from two metal−oxygen (M−O) paths with different interatomic distance and one metal−metal (M−M) path according to the Li_2_MnO_3_ structure were included. The value of the amplitude reduction factor ($S_{0}^{2}$) was obtained from the fitting of the pristine Li_2_MnO_3_ powder and fixed in the fits of the electrode materials.

### 2.5. Online Electrochemical Mass Spectrometry

A customized online electrochemical mass spectrometer (OEMS) with a multi-inlet capillary system (Hiden Analytical, Warrington, UK) was used to analyze the evolving gases, in operando, as a function of the applied potential and temperature. A laboratory-made cell for the OEMS measurement was assembled inside an Argon filled glovebox with Li_2_MnO_3_ as the cathode (ø 10 mm), Li foil as the anode (ø 14 mm), and 100 µL LP 57 as the electrolyte solution. Two polyethylene separators (ø 29 mm) were placed between the cathode and anode. The cell was connected to OEMS through a microcapillary using one of the Swagelok valves located at the head part of the cell. A vacuum of 10^−6^ torr was maintained while sampling the evolved gases at a rate of 12 µL/s. The electrochemical measurements were carried out using VSP-potentiostat (Bio-logic Science instruments, Seyssinet-Pariset, France) in a potential window of 2.4–4.7 V. The variation of desired gases with time was investigated using Mid mode. The measurements were carried out at 0° and 30 °C using thermostats.

## 3. Results and Discussion

### 3.1. Structural and Morphological Characteristics of Li_2_MnO_3_

Figures S1 and S2, respectively, represent the SEM image of the Li_2_MnO_3_ particles measured from the as-prepared annealed product and its XRD profile. It can be seen in Figure S1 that the size of the primary particles varies between 30 and 100 nm, with some forming submicronic and micron-sized aggregates. The XRD pattern in Figure S2 shows peak positions characteristic of the Li_2_MnO_3_ phase that correspond to the *C*2/*m* symmetry, including weak overlapping peaks (020) and (110) at 2ϑ = 20 – 22°, which arose due to the ordering in the transition metal layers comprising Li and Mn ions in an atomic ratio 1:2. This is in accordance with the approach describing the monoclinic Li_2_MnO_3_ structure [67,68] as a layered compound similar to LiMO_2_, thus representing the formula Li_2_MnO_3_ in layered notation as Li[Li_1/3_Mn_2/3_]O_2_, while the ordered [Li_1/3_Mn_2/3_] layers are stacked along the *c*-axis.

**Figure 1:**
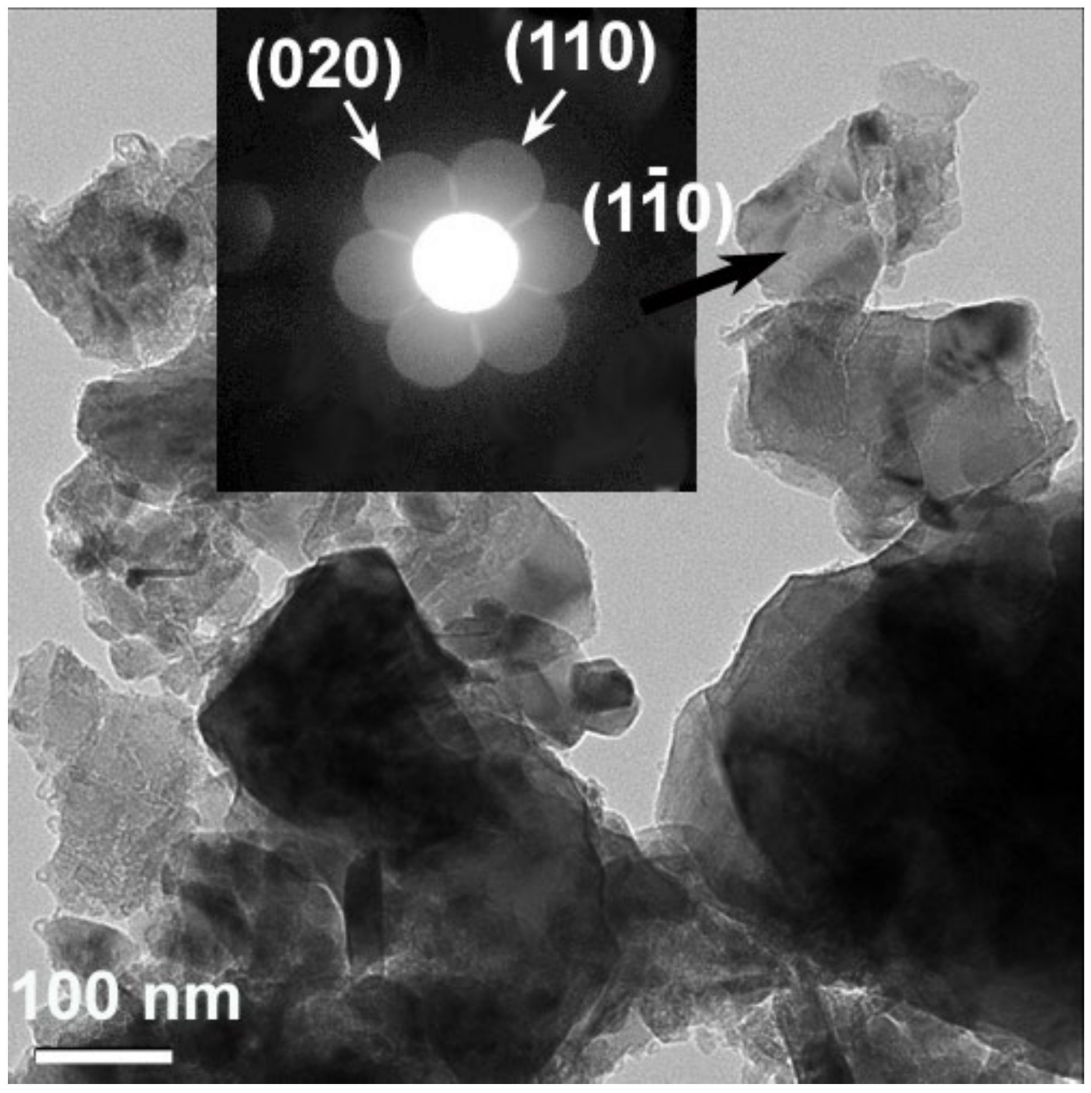
Bright field TEM image of the particles of pristine Li_2_MnO_3_ material. The insert shows an example of CBED pattern, which was taken from the particle indicated by an arrow. The pattern was indexed on the basis of the monoclinic Li_2_MnO_3_ structure.

The unit cell parameters of the monoclinic phase as calculated from the XRD data (reliability factor of R_p_ = 2.55%) are as follows: a = 4.926 Å, b = 8.512 Å, c = 5.022 Å, β = 109.33°, which is in line with the values reported in the literature [68]. The results of TEM analysis are consistent with the structural information obtained by XRD. A TEM image of individual particles of the annealed, as-synthesized material is represented in Figure 1 with an insert showing the CBED pattern, which was taken from the particle indicated with an arrow and indexed on the basis of its monoclinic Li_2_MnO_3_ structure. A small region of this particle was also used to obtain a high-resolution image shown in Figure S3. As expected, the distances measured between the atomic planes were 0.43 nm and 0.41 nm, thus matching, respectively, the interplanar spacings d_020_ and d_110_ in the monoclinic Li_2_MnO_3_ structure. Furthermore, Fourier transform (see insert) of a part of the image looks exactly the same as the CBED pattern (Figure 1) recorded in the diffraction experiment.

### 3.2. Electrochemical Behavior and Structural Evolution of Li_2_MnO_3_ Electrodes at 0 °C, 30 °C, and 45 °C. Impact of their Initial Activation Cycling at 0 °C

**Figure 2:**
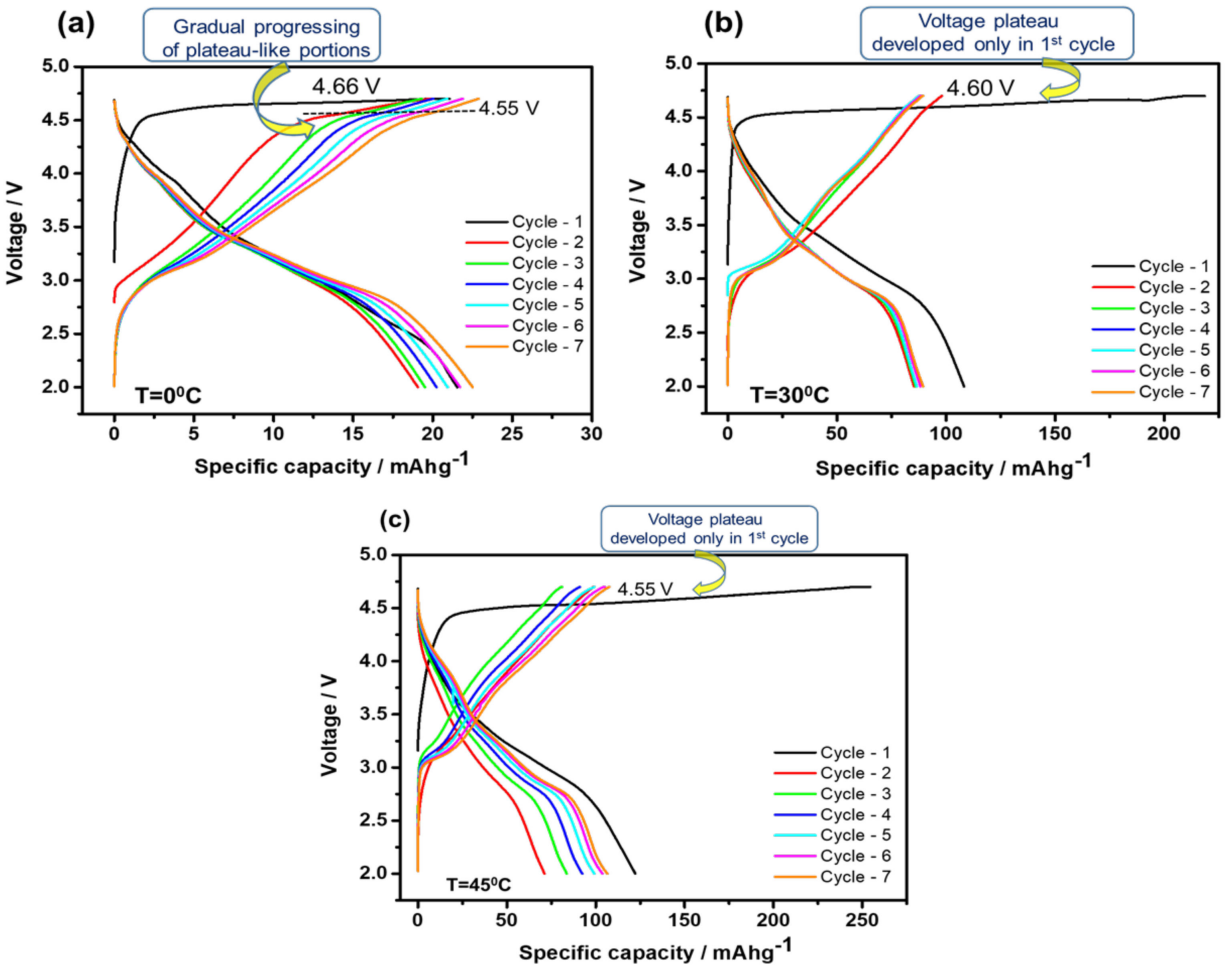
Voltage profiles of Li_2_MnO_3_ electrodes measured during several initial cycles at 0 °C (**a**), 30 °C (**b**), and 45 °C (**c**). The current density applied was 2 mA/g. Potentials of the plateaus registered upon the charging of electrodes to 4.7 V are indicated. At the upper cut-off voltage of 4.7 V, electrodes were kept potentiostatically for 1 h. Note that at 0 °C, plateau-like portions of the charging profiles registered at ~4.55 V developed in a step-wise manner, as indicated in (**a**).

In Figure 2a–c, we compare the voltage profiles measured during the initial galvanostatic charge–discharge cycles from Li_2_MnO_3_ electrodes at 0 °C, 30 °C and 45 °C, respectively. The first charging processes for all of these electrodes up to 4.7 V were characterized by a steep potential increase in the potential range from OCV to ~4.4–4.5 V, at which Li_2_MnO_3_ was electrochemically inactive, followed by an irreversible potential plateau related to the Li_2_MnO_3_ activation and the Li^+^ deintercalation, in agreement with the literature reports [48,49]. It is important to note that upon the initial charge reaction, the potential plateaus were registered at 4.66 V, 4.60 V, and 4.55 V for the above Li_2_MnO_3_ electrodes at 0 °C, 30 °C, and 45 °C, respectively. This observation is in line with our finding in a previous study [41], in which Li_2_MnO_3_-including Li- and Mn-rich electrodes 0.35Li_2_MnO_3_·0.65Li[Mn_0.45_Ni_0.35_Co_0.20_]O_2_ at 0 °C exhibited the 1st charging voltage plateau at ~4.7 V that was 150–200 mV higher compared to those measured at 15 °C, 30 °C, and 45 °C. In addition, only at 0 °C, the charge profiles exhibited short, plateau-like portions at around 4.50–4.55 V, formed continuously during charging to 4.7 V in the 2nd to 5th cycles. This continuously developed plateau-like portion of the charging process at 0 °C, though at lower potential compared to the plateau during the first charge (Figure 2a), seemed to be thermodynamically favorable and may have indicated a continuous activation of Li_2_MnO_3_ and the Li^+^ extraction from the structure. They were gradually progressing, in contrast to the voltage plateaus that developed only in the first charging process of Li_2_MnO_3_ electrodes at 30 °C and 45 °C, shown in Figure 2b,c. In addition, Figure S4, that shows differential capacity plots of the above Li_2_MnO_3_ electrode cycled at 0 °C, also exhibits dQ/dV peaks at potentials close to those of the voltage plateaus.

A similar continuous activation of Li_2_MnO_3_ reflected by gradually developed, plateau-like portions during charging to 4.8 V for the initial five cycles was also observed by Ye et al. [69] in Li-rich Li[Li_1/3–2x/3_Mn_2/3–x/3_Ni_x_]O_2_ (0.09 ≤ x ≤ 0.2) materials doped with minor amounts of nickel. These authors attributed the continuous or stepwise gradual activation of Li_2_MnO_3_ to smaller (optimized) amounts of dopant.

**Figure 3:**
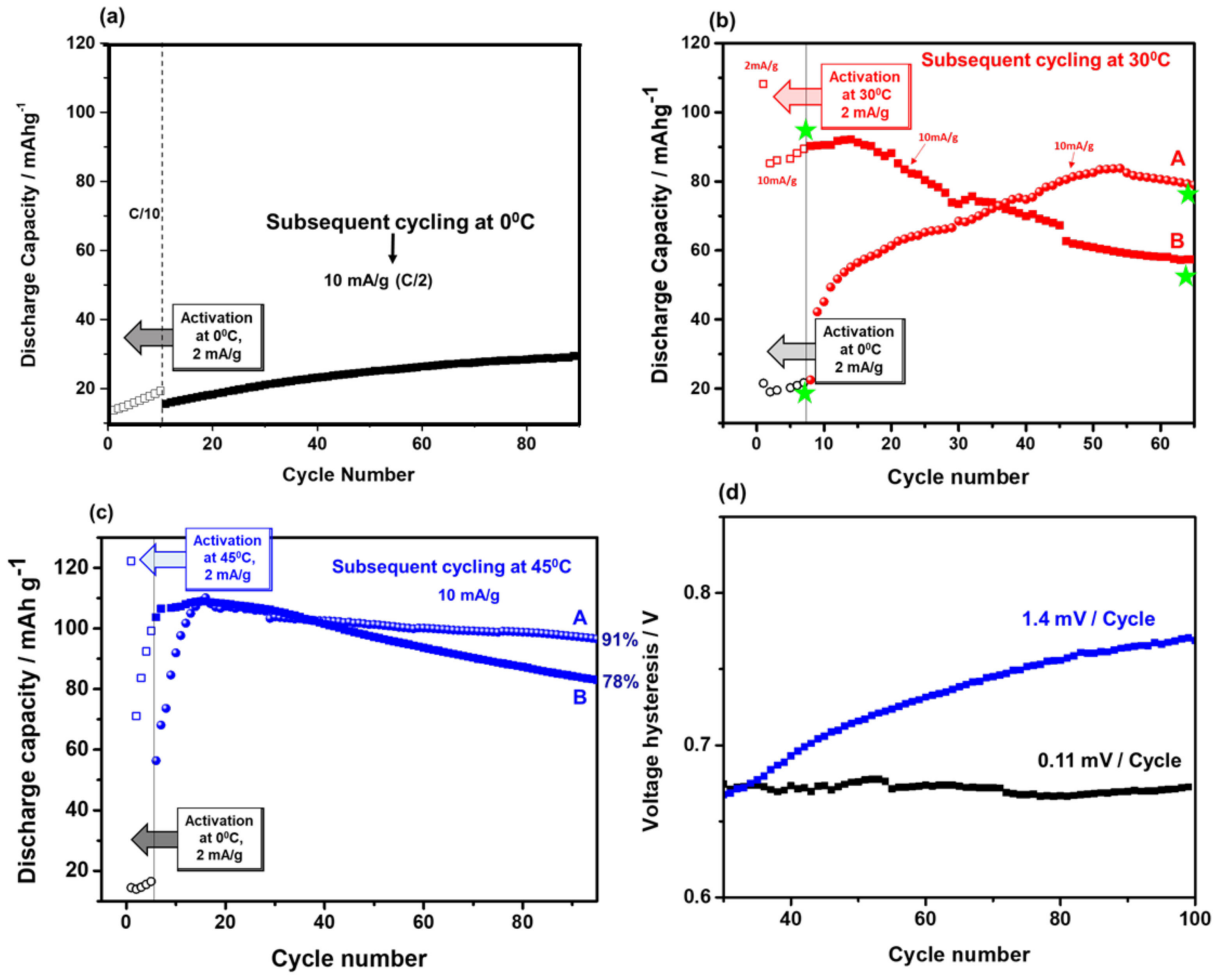
(**a**) Discharge capacity of Li_2_MnO_3_ electrodes activated at 0 °C for several initial cycles (empty symbols). Current density was 2 mA/g corresponded to ~C/10 rate. Subsequent cycles (10 mA/g, ~C/2 rate) at this temperature are also shown (filled symbols). (**b**) Left hand portion: discharge capacity of several initial activation cycles of Li_2_MnO_3_ electrodes at 0 °C (black symbols; 2 mA/g, ~C/10) and at 30 °C (red symbols). In the right hand portion, curve **A** exhibits discharge capacity of Li_2_MnO_3_ electrodes activated at 0 °C and subsequently cycled at 30 °C (10 mA/g, ~C/2). Curve **B** relates to the case when both activation and the subsequent cycling were performed at 30 °C. Note that in both cases of activation at 0 °C or at 30 °C, the subsequent cycling was performed at the same C/2 rate, for the direct comparison. Impedance spectra of Li_2_MnO_3_ electrodes were measured at 30 °C, at 4.4 V upon charging at points marked with green stars. Typical impedance spectra are shown in Figure S6. (**c**) Left hand portion: discharge capacity of several initial activation cycles of Li_2_MnO_3_ electrodes at 0 °C (black circles; 2 mA/g, ~C/10) and 45 °C (blue squares). In the right hand portion, curve **A** exhibits discharge capacity of Li_2_MnO_3_ electrodes activated at 0 °C and subsequently cycled at 45 °C (10 mA/g, ~C/2). Curve **B** relates to the case when both activation and the subsequent cycling were performed at 45 °C. Capacity retention (in %) of these electrodes calculated from 20th to 95th cycles is indicated. (**d**) Black curve displays the voltage hysteresis of Li_2_MnO_3_ electrodes activated at 0 °C for a few initial cycles and subsequently cycled at 45 °C. Blue curve: both activation and the subsequent cycling were performed at 45 °C, as demonstrated in Figure 3c. Changes of the voltage hysteresis per cycle are indicated.

Figure 3a–c shows that the discharge capacities delivered in the initial cycles at 0 °C and 45 °C increased up to ~22 and 100 mAh/g, respectively, while those at 30 °C were stabilized at around 90 mAh/g. We note that the relatively low capacities obtained at 30 °C and 45 °C from electrodes comprising Li_2_MnO_3_ submicronic (partially agglomerated) particles are in line with the values demonstrated in our previous work [58]. It should be noted that a high specific capacity (>200 mAh/g) can be extracted from Li_2_MnO_3_ electrodes only if the active mass comprises nanoparticles. The active mass used herein comprised too big particles, and therefore, the maximal specific capacity that could be demonstrated was 100 mAh/g. However, in this work, the focus is not the optimization of performance in terms of high capacity, but rather, understanding the effect of temperature on the activation process, which is very complicated. The complex process of the Li^+^-ion extraction above 4.5 V from Li- and Mn-rich electrodes (activation) reflected by the potential plateau involves the simultaneous extraction of oxygen from the lattice, and the rearrangement of Mn ions in the case of Li_2_MnO_3_ or TM ions (Mn, Ni, Co) in the case of xLi_2_MnO_3_·(1−x)Li(TM)O_2_ materials. This process can be represented as follows [56,70]:

Li_2_MnO_3_ → 2 Li^+^ + 2e^−^ + MnO_2_ +1/2 O_2_↑ (1)

Recent studies by the Gasteiger’s group using OEMS and HR-TEM [6,70,71] provided strong evidence that oxygen is released not from the bulk Li_2_MnO_3_, but irreversibly from a several nm thick near-surface layer of xLi_2_MnO_3_·(1−x)Li(TM)O_2_ materials. It is important to emphasize that upon charging to high potentials, oxygen can be oxidized to per-oxo species (2O^2−^ ↔ O_2_^2−^ + 2e^−^) and then released as gaseous O_2_ [45,72]. This reversible oxo- (O^2−^) to per-oxo like (O_2_)^n−^ transformation upon charge/discharge of Li-rich oxides is driven by a reductive coupling mechanism, and is still under intensive study [73,74]. As follows from the results in Figure 3a–c, the maximal charge capacities obtained in the 1st cycle at 0 °C, 30 °C, and 45 °C were, respectively 27, 212, and 227 mAh/g. They correspond to ~6, 46, and 49% of the Li-ions extracted from Li_2_MnO_3_, taking into account its theoretical capacity of ~460 mAh/g for 2 Li^+^ extracted per chemical formula. The corresponding discharge capacities delivered in the 1st cycles at these temperatures were 21, 110 and 113 mAh/g, with calculated irreversible capacity losses (ICL) of 1.8, 51, and 52% respectively. The lowest ICL at 0 °C thus implies far fewer undesirable side reactions of the electrode material with traces of HF, PF_5_ (strong Lewis acid) in EC-EMC/LiPF_6_ solution, for instance:

Li_2_MnO_3_(s) + PF_5_(sol) → 2LiF(s) + POF_3_(gas) + MnO_2_(s) (2)

**Figure 4:**
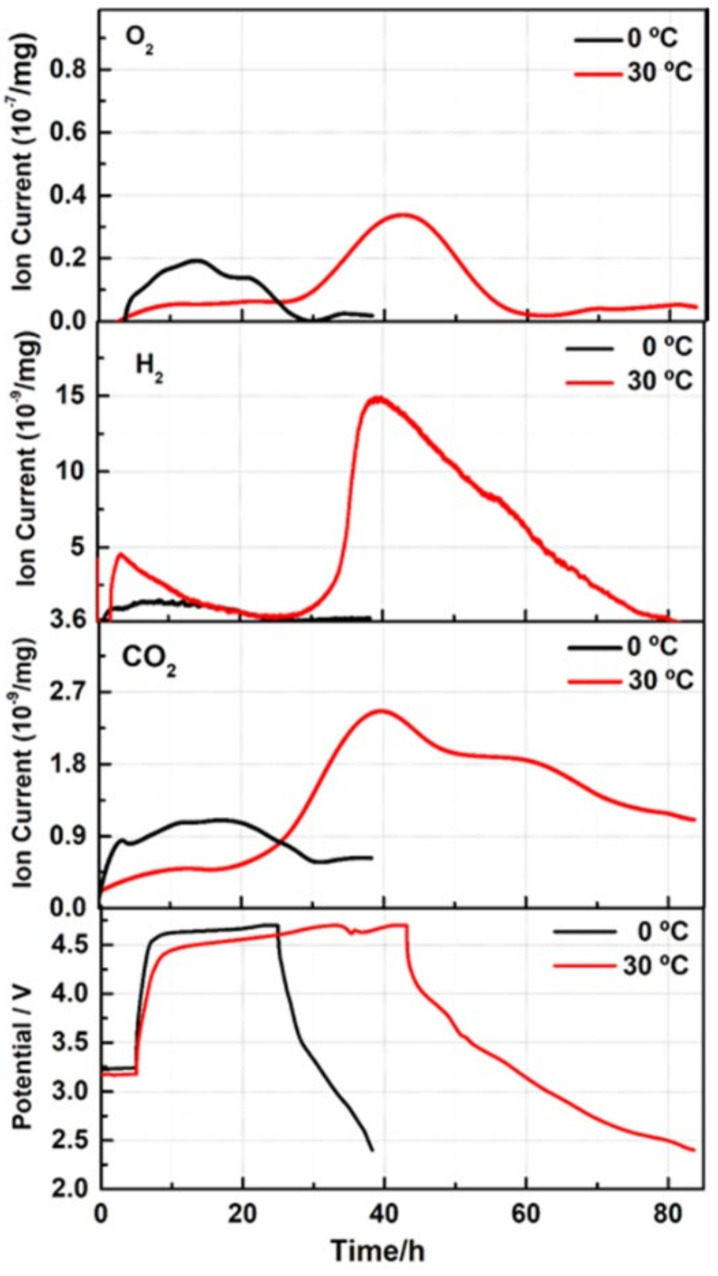
Evolution of gases O_2_, H_2_, and CO_2_ with time during the 1st charge–discharge cycle of Li_2_MnO_3_ electrodes (shown in the bottom chart) measured by OEMS at 0 °C and 30 °C, as indicated. Since during 2nd to 7th initial cycles, less gas was evolved compared to the 1st one, the corresponding ion currents could not be adequately measured because of the instrumental limitations of OEMS. The upper cut-off voltage was 4.7 V in these experiments.

**Figure 5:**
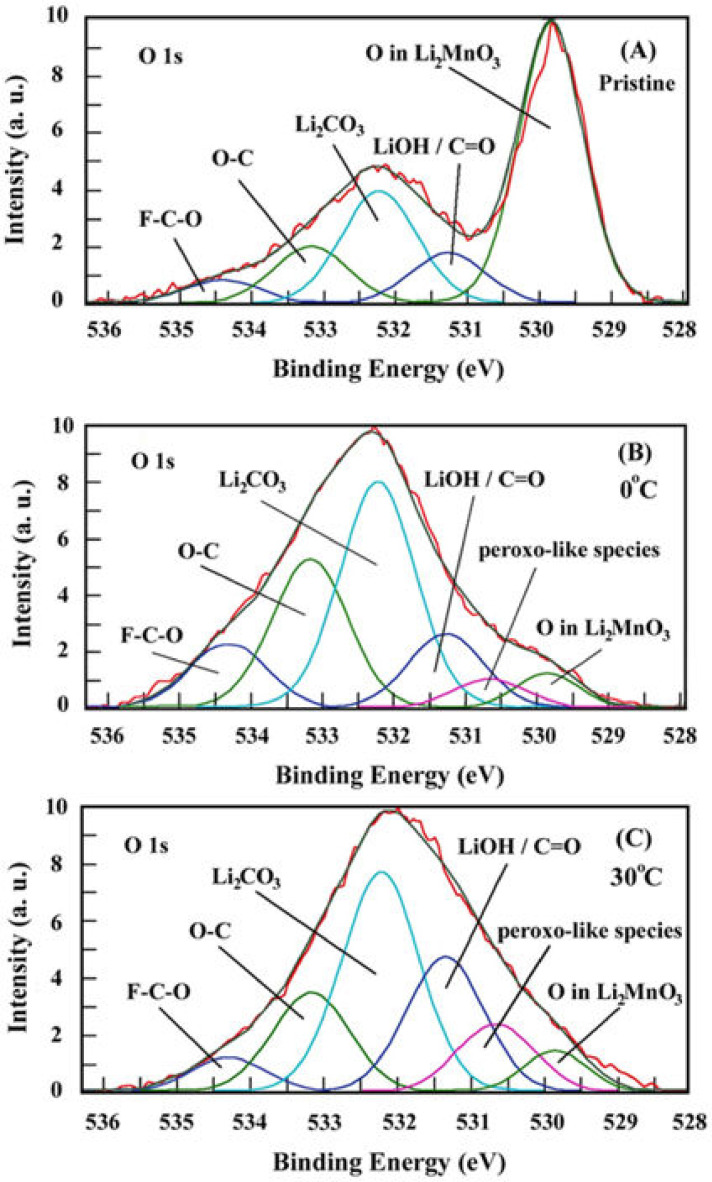
O1s XPS spectra measured from pristine (uncycled) Li_2_MnO_3_ electrode (**A**) and those subjected to the 1st charge–discharge cycles at 0 °C and 30 °C, (**B**) and (**C**), respectively. Voltage profiles of these electrodes are shown in Figure 2a,b.

This may also be related to the reduced interactions of the per-oxo like species formed and the oxygen released at 0 °C with the EC and EMC solvents. (O_2_)^n−^ can promote reactions of the EC absorbed on the electrode surface and its decomposition (by breaking one of the two C–O bonds in the ring) that results in the evolution of CO_2_ and other organic species [75]. Our suggestion on minor interfacial side reactions at 0 °C is consistent with the observed reduced oxygen activity upon charging at this temperature, compared to at 30 °C. This is demonstrated by the results of OEMS studies in Figure 4, and agrees well with the surface analysis of the oxygen XPS spectra of Li_2_MnO_3_ samples subjected to initial cycling at 0 °C and 30 °C (Figure 5).

From XPS measurements, we established that a lower amount (5.2%) of per-oxo like species is formed upon the 1st cycle of Li_2_MnO_3_ electrode at 0 °C compared to that of 11.6% at 30 °C; see Figure 5 and Table S2. This is also evident from the comparative ratio of (lattice oxygen) to (per-oxo species), which was almost twice as high for the latter electrode. Note that the content of (O_2_)^n−^ species calculated from our high-resolution XPS studies agrees well with that number of 9% measured for discharged Li-rich Li_1.2_Ni_0.13_Mn_0.54_Co_0.13_O_2_ materials presented in the work by Tarascon et al. [45]. We suggest that stabilized Li_2_MnO_3_ electrode/solution interface forms during the activation cycles at 0 °C, compared to that at 30 °C and 45 °C, thus providing their stable subsequent cycling performance with higher capacity retention, as demonstrated in Figure 3b,c.

**Figure 6:**
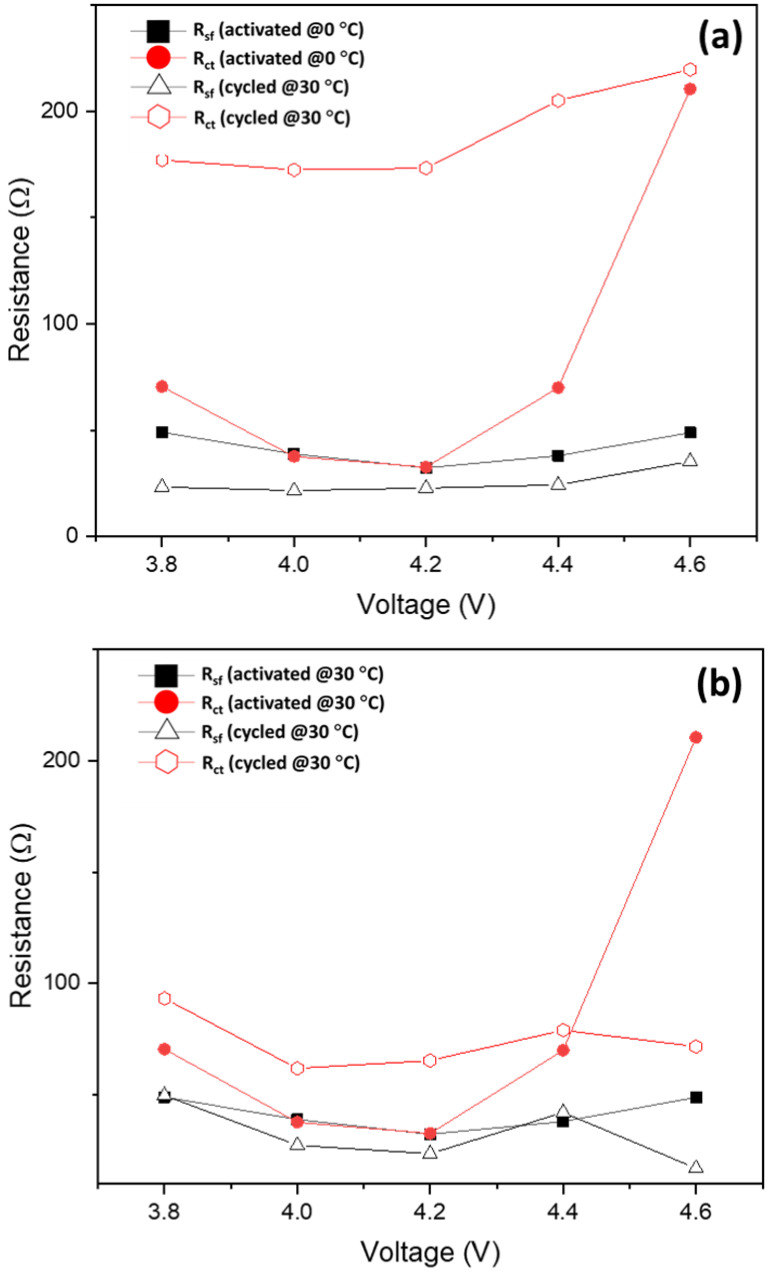
Surface film (R_sf_) and charge-transfer (R_ct_) resistances calculated from impedance spectra measured at 30 °C from Li_2_MnO_3_ electrodes initially activated at 0 °C (**a**) and 30 °C (**b**), respectively, as presented in Figure 3b, curves **A** and **B**. The corresponding points of impedance measurements are marked with green stars in Figure 3b. Typical impedance spectra (Nyquist plots) of these electrodes, measured at 30 °C at 4.4 V upon charging, are displayed in Figure S6.

Clear evidence to support our suggestion comes in the form of the much lower charge-transfer resistance (R_ct_) of Li_2_MnO_3_ electrodes initially activated at 0 °C, compared to at 30 °C, as demonstrated in Figure 6a. This represents the surface film (R_sf_) and charge-transfer resistances of these electrodes as a function of the potential measured during the charging process up to 4.7 V. Figure 6b shows that after 65 cycles, R_ct_ also remained lower for electrodes activated at 0 °C. Only at 4.6 V, R_ct_ increased, probably due to some structural disordering or rearrangements at the electrode surface at high anodic potentials. Note that information on resistances representing the electrochemical processes (as in Figure 6a,b) was obtained from the analysis of the data via Impedance Spectroscopy Genetic Programing. ISGP computes distribution function of relaxation times Γ in the form of Γ plotted vs. log f (f is the frequency range from 100 kHz to 5 mHz used in impedance tests). These plots are shown in the Supplementary Materials Figure S5a–d.

The continuous “activation” of the Li_2_MnO_3_ phase at 0 °C occurring upon charging to 4.7 V at potentials of 4.50–4.55 V was supported by the results of the galvanostatic intermittent titration technique (GITT) measurements, shown in Figure S7. These results confirm that only at 0 °C during the 1st charging process of Li_2_MnO_3_, the variation with time of the transient electrode potential exhibited short, plateau-like regions at 4.6 V, followed by an extended plateau, similar to that in Figure 2a. Thus, we consider the activation of Li_2_MnO_3_ electrodes at 0 °C to be a step-by-step process that was not detected in the initial cycles at the higher temperatures of 30 °C and 45 °C. A few questions, therefore, can be addressed, like: What is the possible impact of this step-by-step activation during several initial cycles at 0 °C on the subsequent electrode cycling at this temperature and at higher temperatures? What is its effect on the capacity fading, on the evolution of the voltage hysteresis and on electrode impedance? From the typical plot of the discharge capacity delivered by the Li_2_MnO_3_ electrode activated at 0 °C and subsequently cycled at this temperature (Figure 3a), it is clearly seen that after the activation cycles, the capacity increases smoothly, reaching maximal values of ~25–30 mAh/g. In contrast, 3–4 times greater capacities can be obtained when Li_2_MnO_3_ is activated at 0 °C and cycled at higher temperatures, i.e., 30 °C or 45 °C, as demonstrated by the cycling profiles (curves **A**) in Figure 3b,c, respectively. These results demonstrate that only when a few initial cycles were performed at a low temperature, providing step-by-step activation, the capacity increased substantially, and the capacity fading was less and the voltage hysteresis was considerably mitigated and stabilized upon cycling at a higher temperature (45 °C), as demonstrated in Figure 3d. This is in line our previous findings [41], i.e., that the activation of Li- and Mn-rich cathodes (0.35Li_2_MnO_3_·0.65Li[Mn_0.45_Ni_0.35_Co_0.20_]O_2_) at low temperature results in an increased discharge capacity, decreased average charge voltage, and voltage hysteresis. As we noted in that paper, the results demonstrated by low temperature activation (“low-T activation”) of the above cathodes were similar to those shown by other groups using potential window opening or step-wise increasing anodic potential limits [15,45,76,77,78]. For instance, increased discharge capacity and decreased fading were observed by Sato et al. [78] and Ohsawa et al.[79], when cells with Li[Ni_0.17_Li_0.2_Co_0.07_Mn_0.56_]O_2_ cathodes were cycled through step-wise precycling treatment, which included increasing the upper potential limit by 0.1 V from 4.5 V every two cycles to 4.8 V. This precycling treatment means reaching and traversing the voltage plateau several times, after which the Li-ions are extracted and the oxygen is released from the material. Dahn et al. [80] also came to a conclusion that a repeated stepwise traverse of the oxygen-release plateau resulted in higher cycling capacities of Li- and Mn-rich Li[Li_1/5_Ni_1/5_Mn_3/5_]O_2_ cathodes in Li-cells, compared to one-step oxygen release. By analyzing the literature data and the results obtained in the present work for Li_2_MnO_3_ electrodes activated at 0 °C, we assume that this “low-T activation” indeed provided a useful repeated stepwise traversing of the voltage plateau at >4.5 V over several cycles (from 2nd to 5th); see Figure 2a. These short, plateau-like portions were developed in a step-wise manner from the same cycling procedure upon charging Li_2_MnO_3_ to 4.7 V. The effect of the “low-T activation” was that the capacity of Li_2_MnO_3_ electrodes increased and leveled-off upon further cycling at 30 °C, in contrast to those activated in one-step at 30 °C and cycled at this temperature (Figure 3b, curves **A** and **B**, respectively). Stepwise activation at 0 °C also pronouncedly affected the further cycling of Li_2_MnO_3_ at 45 °C, resulting in a stabilized performance after “low-T activation”, as reflected by higher capacity retention and much lower and stabilized the voltage hysteresis; see Figure 3c,d, respectively. These findings are crucially important, since capacity fading and large voltage hysteresis are among the main problems which need to be resolved for Li- and Mn-rich cathode materials in advanced LIBs [2,4,5]. It is important to note that the effect of the “low-T activation” of Li_2_MnO_3_ discussed above confirms the hypothesized mechanism behind the improved electrochemical behavior of 0.35Li_2_MnO_3_·0.65Li[Mn_0.45_Ni_0.35_Co_0.20_]O_2_ materials activated at 0 °C in our earlier work [41]. This mechanism suggested a gradual, step-wise activation of these electrodes at 0 °C, allowing slower structural activation/reorganization of the Li_2_MnO_3_ component to occur. This, in turn results in a greater degree of the TM-layer being accessible for Li^+^ ion extraction/re-insertion and a smaller amount of the surface oxygen release. Indeed, as was confirmed by our OEMS and XPS studies of Li_2_MnO_3_ electrodes activated at 0 °C, the amounts of O_2_ evolved and per-oxo species formed were lower compared to those at 30 °C (Figure 4 and Figure 5, respectively). Below, we present the results of some structural studies of Li_2_MnO_3_ electrodes initially activated at 0 °C and 30 °C.

### 3.3. Structural Aspects of the Initial Cycling of Li_2_MnO_3_ Electrodes at 0 °C and 30 °C

**Figure 7:**
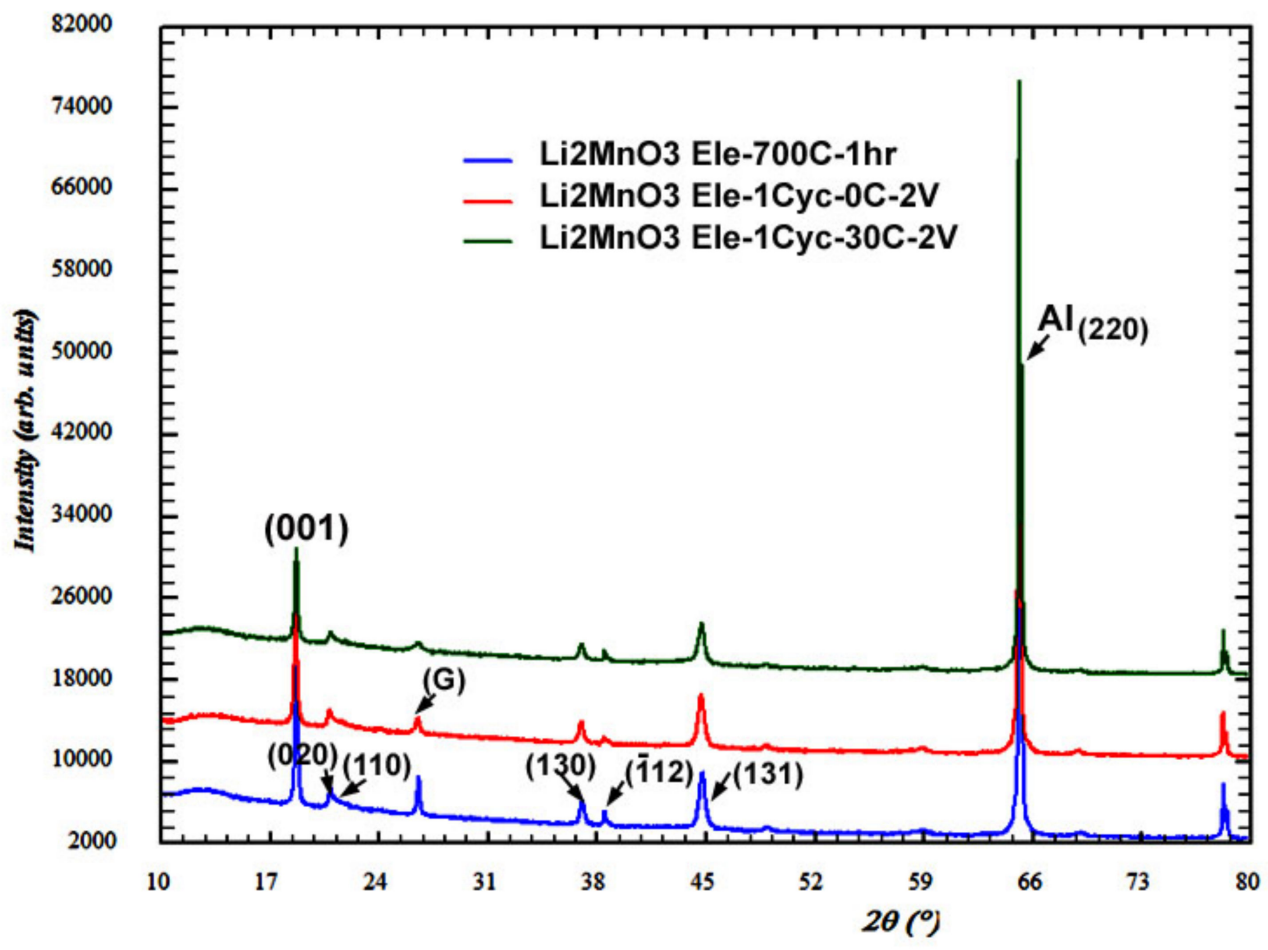
Comparison of XRD patterns recorded from Li_2_MnO_3_ electrodes on the Al-foil current collectors. Label G is related to a graphite peak, the peak at 2ϑ = 65.1° is associated with (220) reflection from Al-foil. Blue, red and green profiles correspond, respectively, to as-prepared (annealed) powder and to electrodes subjected to the first charge–discharge cycle at 0 °C and 30 °C.

It is evident from the voltage profiles of Li_2_MnO_3_ electrodes initially cycled at 0 °C, 30 °C, and 45 °C (Figure 2a–c) that electrochemical activities were developed at around 3 V. We propose that these electrochemical processes reflect the formation of spinel or spinel-like phases as a result of the layered-to-spinel transformation which is typical for Li(TM)O_2_ cathode materials during charge–discharge cycling [28,35,49]. Figure 7 compares the XRD patterns of an uncycled (pristine) Li_2_MnO_3_ electrode with those of structurally identical electrodes initially charged to 4.7 V and discharged at 2.0 V at 0 °C and 30 °C, respectively.

**Figure 8:**
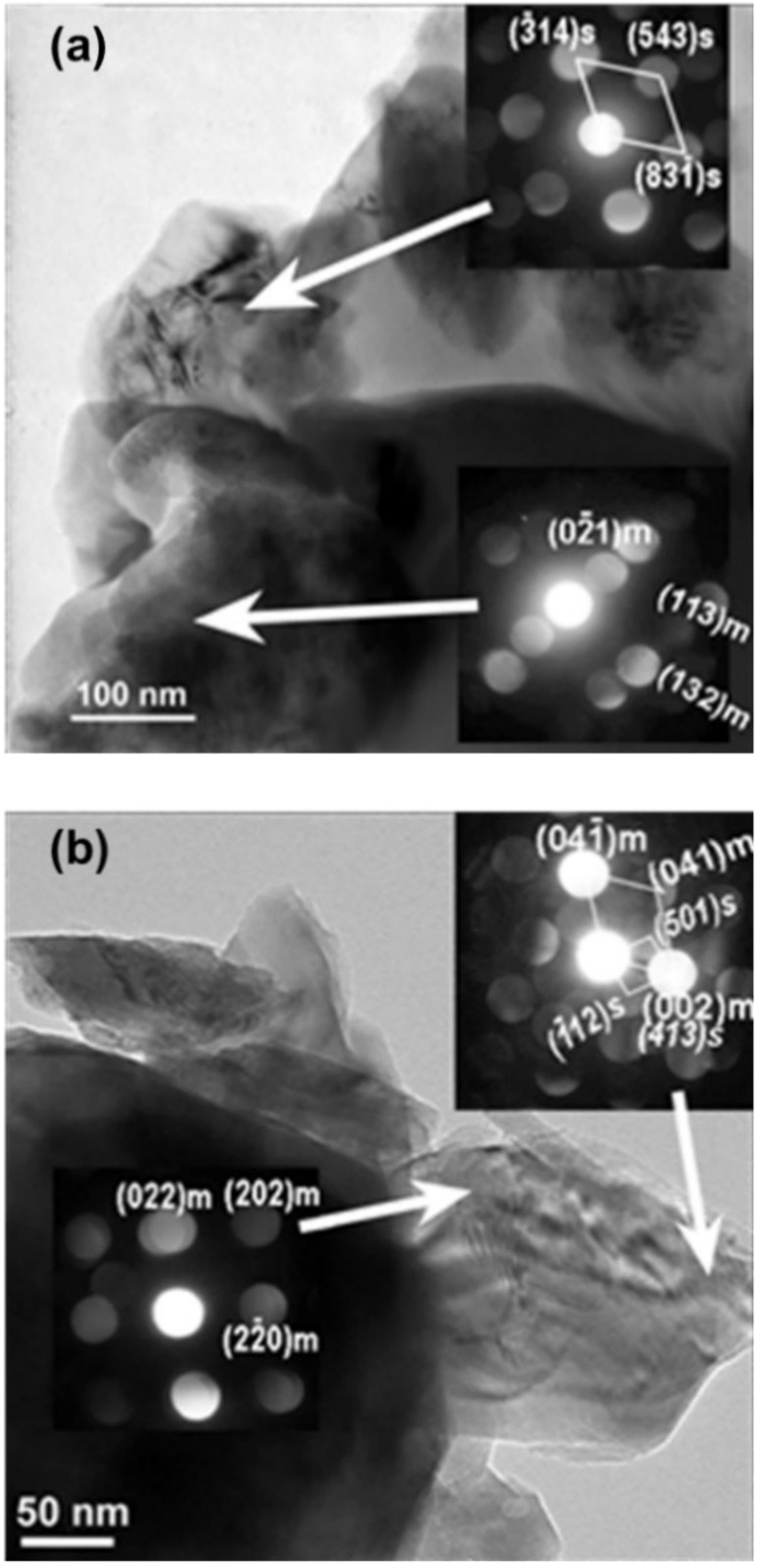
(**a**) TEM micrograph of Li_2_MnO_3_ electrode after the first charge/discharge cycle at 0 °C. The inserts show CBED patterns, which were taken from grains indicated by arrows. The pattern in the bottom insert is indexed on the basis of the monoclinic Li_2_MnO_3_ phase (m), and the pattern in the upper insert demonstrates the presence of the tetragonal spinel Li_2_Mn_2_O_4_ (s). (**b**) TEM micrograph of Li_2_MnO_3_ electrode after the first charge/discharge cycle at 30 °C. The inserts show CBED patterns taken from grains indicated by arrows. The bottom pattern is indexed to the monoclinic Li_2_MnO_3_ phase (labeled m), and the pattern in the upper insert contains two systems of reflections related to the monoclinic phase (m) and to the tetragonal spinel (s).

As follows from Table S1, the unit cell parameters of cycled Li_2_MnO_3_ only slightly changed compared to those of the pristine electrode. The XRD patterns recorded from the cycled electrodes did not show any new peaks in addition to the peaks of the monoclinic phase. Nevertheless, the TEM examinations revealed that the tetragonal spinel Li_2_Mn_2_O_4_, described by space group *I4amd* [81], was formed in electrodes even during the first cycle, both at 0 °C and at 30 °C. The cell parameters of Li_2_Mn_2_O_4_ were calculated as follows: a = 17.540 Å and c = 8.205 Å. The corresponding TEM micrographs taken from samples subjected to the first cycle at 0 °C and at 30 °C are shown, respectively, in Figure 8a,b. The inserts in these figures represent the convergent-beam electron diffraction (CBED) patterns, which were taken from the grains indicated by arrows. The bottom inserts correspond to the main monoclinic Li_2_MnO_3_ phase, and the upper inserts contain patterns indexed in terms of the tetragonal spinel Li_2_Mn_2_O_4_. The upper insert in Figure 8b also contains an additional reflection system associated with the monoclinic phase (m). The high-resolution image in Figure S8 is an additional illustration of the presence of tetragonal spinels in the Li_2_MnO_3_ electrode after the first charge–discharge cycle. This image was taken from a grain related to the upper insert in Figure 8a. As expected, the corresponding Fourier transformed electron diffraction data were indexed to the tetragonal spinel Li_2_Mn_2_O_4_ in full accordance with the CBED pattern recorded from this grain.

**Figure 9:**
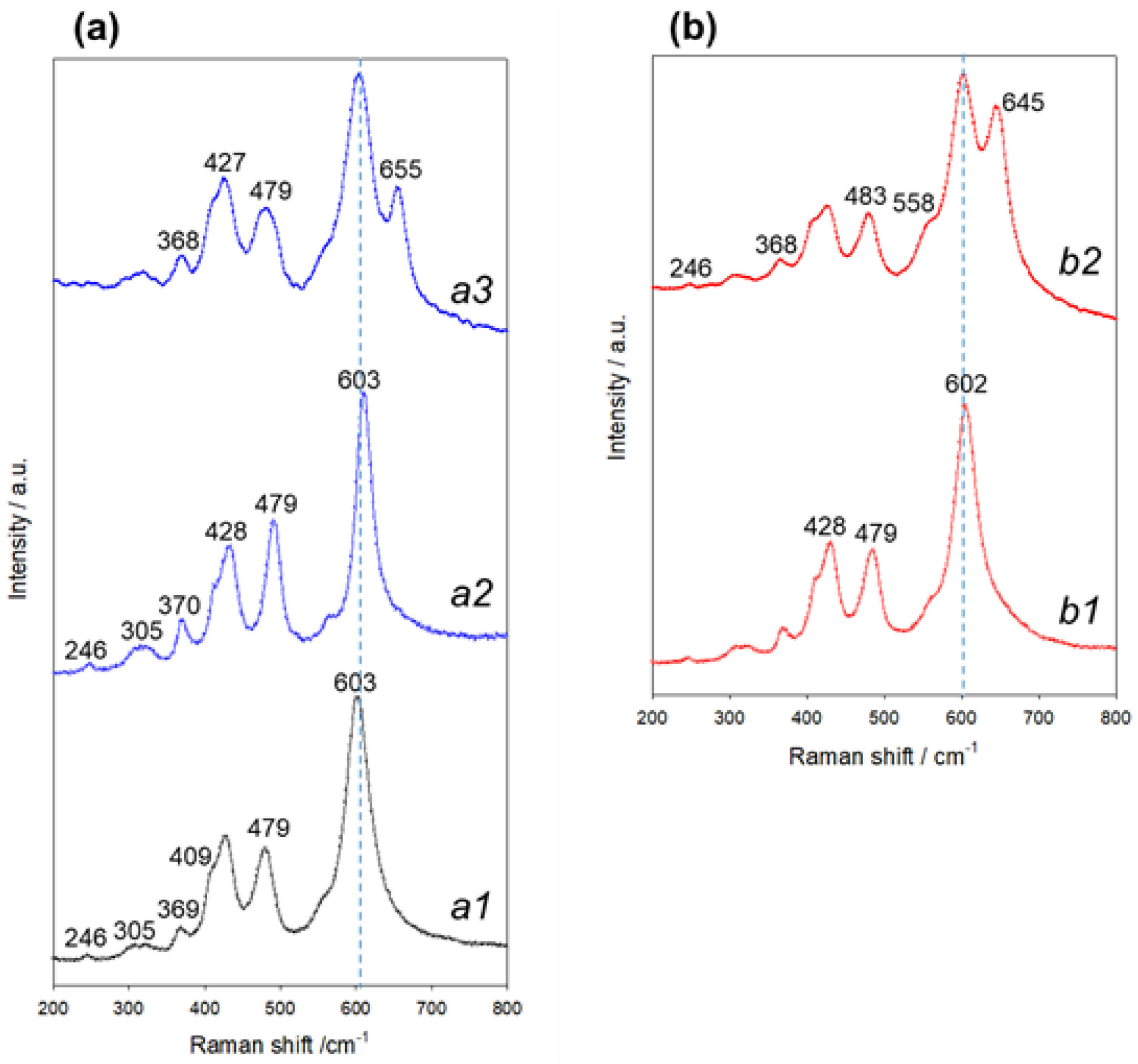
(**a**) Typical Raman spectra of pristine (uncycled) Li_2_MnO_3_ electrode (*a1*) and of a similar electrode after the 1st charge–discharge cycle at 0 °C measured at arbitrary locations (*a2*) and (*a3*); (**b**) spectra related to Li_2_MnO_3_ electrode after the 1st charge–discharge cycle at 30 °C measured at arbitrary locations (*b1*) and (*b2*).

The results of the electron-diffraction studies demonstrating the formation of the spinel phase after the first cycle were supported by Raman spectroscopy measurements. Figure 9 exhibits a typical Raman spectrum of a pristine Li_2_MnO_3_ (uncycled) electrode, showing several well-resolved peaks (bands) at 603, 560, 428, 409, 369, 305, and 246 cm^−1^. These are in agreement with the literature reports on the monoclinic Li_2_MnO_3_ phase [67]. However, the Raman responses of electrodes subjected to a first cycle show some changes, namely a decreased intensity of the main peak at 603 cm^−1^, its asymmetry and broadening related to a cationic disorder, and increasing the amount of stacking faults [82].

We suggest that at anodic potentials >4.5 V, the Li^+^-ions can be further extracted from the LiMn_2_O_4_ phase formed due to the layered-to-spinel transition (as demonstrated by electron diffraction studies, Figure 8a,b), providing emerging of new Raman bands at wavenumbers ~655 and 645 cm^−1^ in the spectra of Li_2_MnO_3_ electrodes cycled at 0 °C and 30 °C, respectively [83]. Upon the discharging process, Li^+^ ions can be inserted into the cubic spinel species to form the tetragonal phase according to the following reaction:

LiMn_2_O_4_ + e^−^ + Li^+^ → Li_2_Mn_2_O_4_ (3)

**Figure 10:**
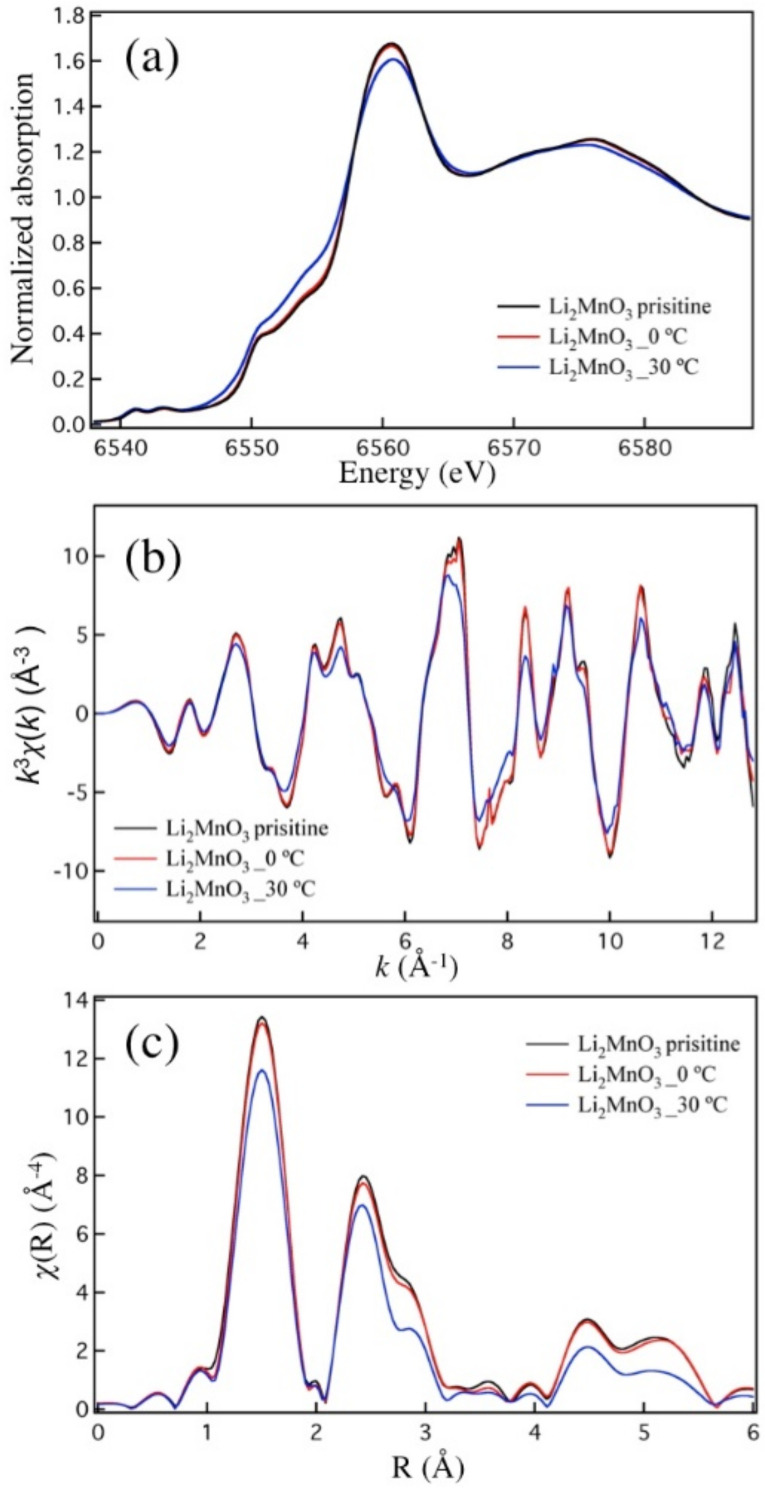
(**a**) Normalized Mn K-edge XANES spectra of Li_2_MnO_3_ samples: pristine powder and electrodes after the 1st cycle (charge to 4.7 V, discharge to 2.0 V) at 0 °C and 30 °C, as indicated. The voltage profiles of these electrodes are shown in Figure 2a,b, respectively. (**b**) k^3^-weighted EXAFS χ(k) of Li_2_MnO_3_ samples: pristine powder and electrodes after the 1st cycle (charge to 4.7 V, discharge to 2.0 V) at 0 °C and 30 °C, as indicated. The voltage profiles of these electrodes are shown in Figure 2a,b, respectively. (**c**) Fourier Transform magnitude of k^3^-weighted EXAFS data (|χ(R)|) of Li_2_MnO_3_ samples (k-range: 2.5-11 Å^−1^; R_bkg_:1.25 Å).

An important piece of information on structural bulk and surface changes of Li_2_MnO_3_ electrodes subjected to initial charge–discharge cycles at 0 °C and 30 °C was obtained from Extended X-ray Absorption Fine Structure – X-ray Absorption Near Edge Structure (EXAFS-XANES) and high-resolution XPS studies. As seen in Figure 10a, the XANES data of the Li_2_MnO_3_ electrode cycled at 0 °C were almost identical to those of the as-prepared pristine material, which indicates the same oxidation state of Mn in these samples. Note that along with Mn^4+^, some Mn^3+^ ions may also be present in the disordered surface layer of Li_2_MnO_3_ electrodes subjected to charge–discharge cycle. This is in accordance with an analysis of the magnetic characteristics of these electrodes at various states-of-charge, as discussed in [84]. In addition, one should take into account that Mn^3+^-containing species (LiMnO_2_) can be formed upon oxygen release from the surface of Li_2_MnO_3_. Moreover, as shown in a recent work [85], based on the calculation of Bader charges and magnetic moments, the Mn oxidation state in HE-NCM can be reduced in the presence of per-oxo like (O_2_)^2−^ as a result of anionic charge overcompensation. The EXAFS data of Li_2_MnO_3_ cycled at 0 °C and 30 °C (Figure 10b,c, respectively) have similar features to the pristine Li_2_MnO_3_. The major change in the electrode cycled at 30 °C was observed as the reduced amplitude in the EXAFS data, probably because of the structural disorder near the Mn sites. From the fitting results (Table S3 and Figure S9) of the EXAFS data, we conclude that an increase occurred in the obtained Debye-Waller factors for Mn-O1 and Mn-Mn paths in the Li_2_MnO_3_ sample cycled at 30 °C, compared with the as-prepared material and the cathode material cycled at 0 °C. Such changes in the Debye-Waller factors were very likely due to structural disorder near the Mn sites, which may be the main reason for the reduction of the amplitude of Mn-O1 and Mn-Mn peaks in the Li_2_MnO_3_ electrode after charge–discharge at 30 °C.

High-resolution XPS data of the above samples also demonstrate that in Li_2_MnO_3_ electrodes subjected to initial cycling at 0 °C and 30 °C, some surface species are formed with the Mn-oxidation states both of 3+ and 2+. This is evident from comparing the XPS spectra of manganese. The Mn 2p structure of cycled samples (Figure S10a) was slightly shifted to lower energy and highly broadened at its lower-energy side, raising the possibility of the formation of additional phases with Mn valences lower than 4+ in pristine (uncycled) Li_2_MnO_3_. This was indeed confirmed by the measured Mn 3s structure, that demonstrated a peak splitting (Δ, eV) higher than that of Li_2_MnO_3_, as illustrated in Figure S10b. For the pristine sample Δ~4.50 eV, in contrast to initially cycled Li_2_MnO_3_ at 0 °C and 30 °C, the peak splitting values were estimated from XPS spectra as Δ~5.60 and Δ~6.30 eV, respectively corresponding to the Mn^3+^ and Mn^2+^ species formed on the surface of the Li_2_MnO_3_ particles [86].

## 4. Conclusions

We established in this work that initial (activation) cycling of Li_2_MnO_3_ electrodes performed at 0 °C results in increased discharge capacity, higher capacity retention, and decreased and stabilized voltage hysteresis upon subsequent cycling at higher temperatures, i.e., 30 °C or 45 °C. This is due to the fact that at 0 °C, Li_2_MnO_3_ electrodes undergo step-by-step activation, providing stepwise traversing of the voltage plateau at >4.5 V over several initial cycles. Importantly, activation cycling performed at 30 °C or 45 °C is characterized by voltage plateaus at 4.60 and 4.55 V, respectively, that develop only in the first charging process of Li_2_MnO_3_ electrodes; in contrast to 0 °C activation, this leads to considerable capacity fading and substantially increased voltage hysteresis in further cycling. These findings are in accordance with those reported by us previously [41]: The activation of 0.35Li_2_MnO_3_·0.65Li[Mn_0.45_Ni_0.35_Co_0.20_]O_2_ materials comprising Li_2_MnO_3_ as the main component at 0 °C leads to extra capacity of these electrodes. This results in an increased discharge capacity, decreased average charge voltage, and lower voltage hysteresis. We conclude that the irreversible capacity loss of Li_2_MnO_3_ electrodes activated at 0 °C is relatively low. The amount of per-oxo like species formed and the oxygen, H_2_, and CO_2_ released during activation at low temperature were also lower compared to cathodes activated at 30 °C and 45 °C. This indicates the occurrence of fewer interfacial side reactions and the formation of an improved surface passivation at 0 °C. The considerably lower charge-transfer resistance of these electrodes, calculated from impedance spectra measured upon charging to 4.7 V, also supports this conclusion. We furthermore conclude that Li_2_MnO_3_ preserved its bulk structure upon activation cycles at 0 °C and 30 °C, with only some structural disorder near the Mn-sites (at 30 °C) and partial transformation (at the particle surface) from the layered-type ordering to a tetragonal spinel Li_2_Mn_2_O_4_ phase, which is typical for these systems.

We believe that the present work on initial (activation) cycling of Li_2_MnO_3_ at 0 °C resulting in improved behavior when continuing operation at higher temperature is important for understanding the complex activation mechanism of high specific capacity Li- and Mn-rich NCM cathode materials for advanced Li-ion batteries.

## Supplementary Materials

The following are available online at https://www.mdpi.com/1996-1944/13/19/4388/s1. Figure S1: Scanning Electron Microscopy image of the as-prepared Li_2_MnO_3_ annealed consequently at 400° and 700 °C for 1 h at each temperature, under air. Figure S2: XRD pattern collected from the as-prepared Li_2_MnO_3_ powder, which was annealed consequently at 400° and 700 °C for 1 h at each temperature, under air. Figure S3: High-resolution image taken from the particle indicated by the arrow in Figure 1. The distances between the atomic planes of 0.43 nm and 0.41 nm match, respectively, the interplanar spacings d_020_, and d_110_ in the monoclinic Li_2_MnO_3_ structure. The Fourier transform in the insert looks exactly the same as the CBED pattern in the insert in Figure 1. Figure S4: Differential capacity dQ/dV profiles of Li_2_MnO_3_ electrodes measured during several initial cycles at 0 °C (a), 30 °C (b), and 45 °C (c) as shown in Figure 3. Figure S5. The DFRT for impedance of Li_2_MnO_3_ electrodes measured at 30 °C after initial activation cycles performed at 0 °C (a) and after subsequent cycling at 30 °C (b), and for similar Li_2_MnO_3_ electrodes measured at 30 °C after initial activation cycles performed at 30 °C (c) and after subsequent cycling at the same temperature of 30 °C (d) Peaks correspond to surface film (sf) and charge-transfer (ct) resistances. Typical impedance spectra of Li_2_MnO_3_ electrodes measured at 30 °C at 4.4 V upon charging are shown in Figure S6. Typical impedance spectra (Nyquist plots) of Li_2_MnO_3_ electrodes measured at 4.4 V upon charging, at 30 °C. Four points of impedance measurements are marked with green stars in Figure 3b, namely: (a) after activation cycles at 0 °C; (b) after activation cycles at 0 °C with subsequent cycling at 30 °C; (c) after activation cycles at 30 °C; (d) after activation cycles at 30 °C with subsequent cycling at 30 °C. Figure S7 Variations with time of the transient electrode potential measured during the 1st charging process by three subsequent GITT titration stages of Li_2_MnO_3_ samples at 0 °C (a) and 30 °C (b). Coin-type cells, LP-57 solution. The current density applied was ~4 µA/cm^2^ corresponding to ~2 mA/g. Semi-filled, filled, and empty symbols relate to OCV potentials of 4.10 V, 4.36 V, and 4.42 V in (a) and 4.33 V, 4.35 V, and 4.36 V in (b), respectively. Figure S8. High-resolution image taken from the particle shown in Figure 7a. This particle was identified as the tetragonal spinel Li_2_Mn_2_O_4_. The indexed Fourier transform shown in the insert is in accordance with the corresponding CBED pattern in Figure 7a. Figure S9. Mn K-edge Fourier transform magnitudes of k^3^-weighted EXAFS data and theoretical fits of Li_2_MnO_3_ materials: pristine powder (a) and electrodes after the 1st cycle at 0 °C (b) and at 30 °C (c). Figure S10. Mn 2p and Mn 3s XPS spectra, (a) and (b), respectively measured from uncycled (pristine) Li_2_MnO_3_ electrode (red lines) and those subjected to the first charge-discharge cycles at 0 °C and 30 °C (green and blue lines, respectively). The Mn 3s spectra include color-coded values for the magnitude of the splitting Δ, eV between the two peaks. Voltage profiles of these Li_2_MnO_3_ electrodes are shown in Figure 2a,b. Table S1. Cell parameters of Li_2_MnO_3_ materials calculated from the corresponding XRD patterns. Table S2. Results of XPS measurements of the lattice oxygen in Li_2_MnO_3_, per-oxo like and other components as calculated by fitting of the corresponding O 1s spectra measured from pristine (uncycled) Li_2_MnO_3_ electrode and those after initial activation cycles at 0° and 30 °C. The corresponding binding energies (in eV) are shown in blue color. Table S3. Best fit results for the structural parameters obtained by analysis of the Mn K-edge EXAFS data of Li_2_MnO_3_ samples: pristine powder and electrodes after the 1st cycle (charge to 4.7 V, discharge to 2.0 V) at 0° and 30 °C. N: coordination number; R: interatomic distance; σ^2^: Debye-Waller factor (mean-square disorder in R); ∆E_o_: Energy shift; (amp): the amplitude reduction factor.
