# Supplementary material for: Electrochemical Activation of Li2MnO3 Electrodes at 0 °C and Its Impact on the Subsequent Performance at Higher Temperatures
Source: Materials (Basel). 2020 Oct 1;13(19):4388. doi: 10.3390/ma13194388 (PMC7579396; doi:10.3390/ma13194388)
Supplement: Supplementary file 1 [file materials-13-04388-s001.pdf]

materials

Supporting Information

The Supporting Information contains:

SEM and HR-TEM images; XRD pattern of the as-prepared Li_2MnO_3; differential capacity plots of Li_2MnO_3 electrodes measured during several initial cycles at 0 °C, 30 °C, and 45 °C; typical impedance spectra of these electrodes (30 °C, 4.4 V); decsription of the impedance spectroscopy genetic programing (ISGP) used for the analysis of these spectra; results of galvanostatic intermittent titration technique (GITT) of Li_2MnO_3 electrodes during the 1st cycle at 0 °C and 30 °C. Results of structural and surface analysis by EXAFS and XPS of pristine Li_2MnO_3 and electrodes after the 1st cycle at 0 °C and at 30 °C are also present in Figures S9 and S10, and Tables S2 and S3.

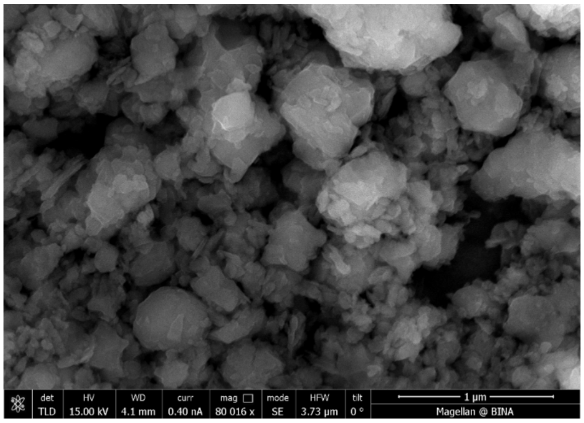

Figure S1. Scanning Electron Microscopy image of the as-prepared Li_2MnO_3 annealed consequently at 400 °C and 700 °C for 1 h at each temperature, under air.

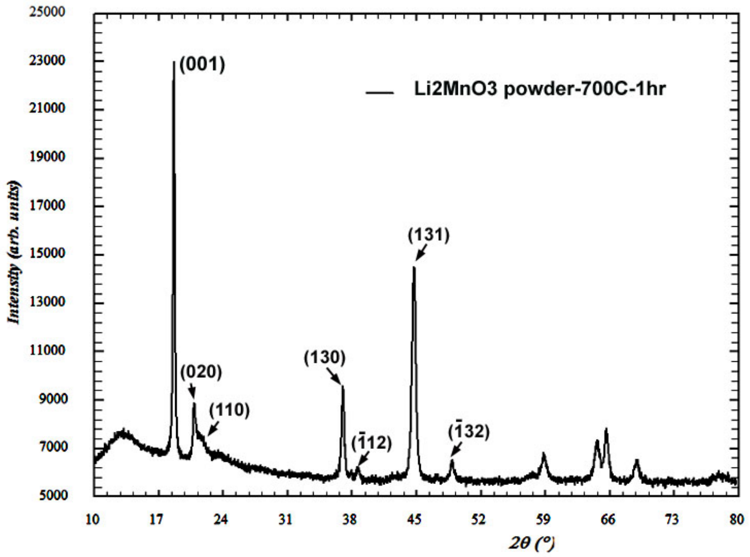

Figure S2. XRD pattern collected from the as-prepared Li_2MnO_3 powder, which was annealed consequently at 400 °C and 700 °C for 1 h at each temperature, under air.

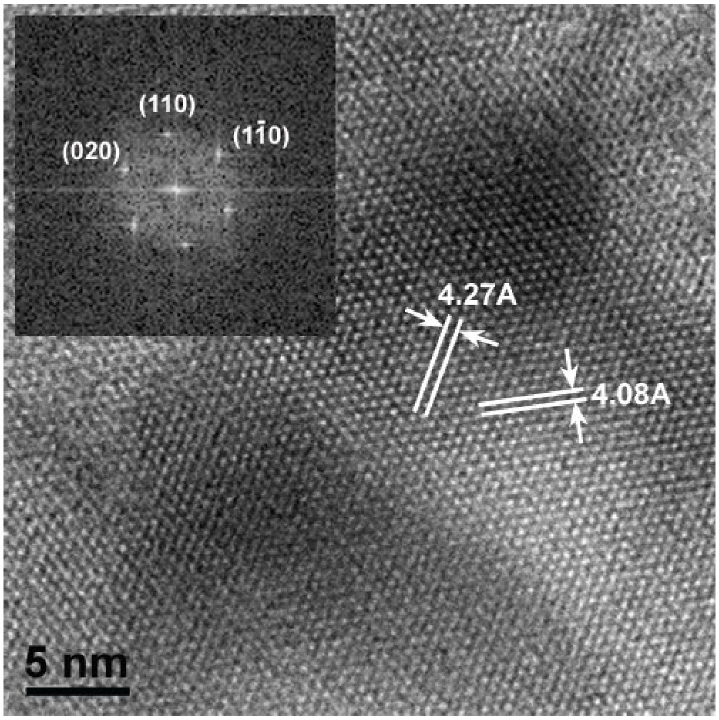

Figure S3. High-resolution image taken from the particle indicated by the arrow in **Figure 1.** The distances between the atomic planes of 0.43 nm and 0.41 nm match, respectively, the interplanar spacings d_{020}, and d_{110} in the monoclinic Li_2MnO_3 structure. The Fourier transform in the insert looks exactly the same as the CBED pattern in the insert in **Figure 1.**

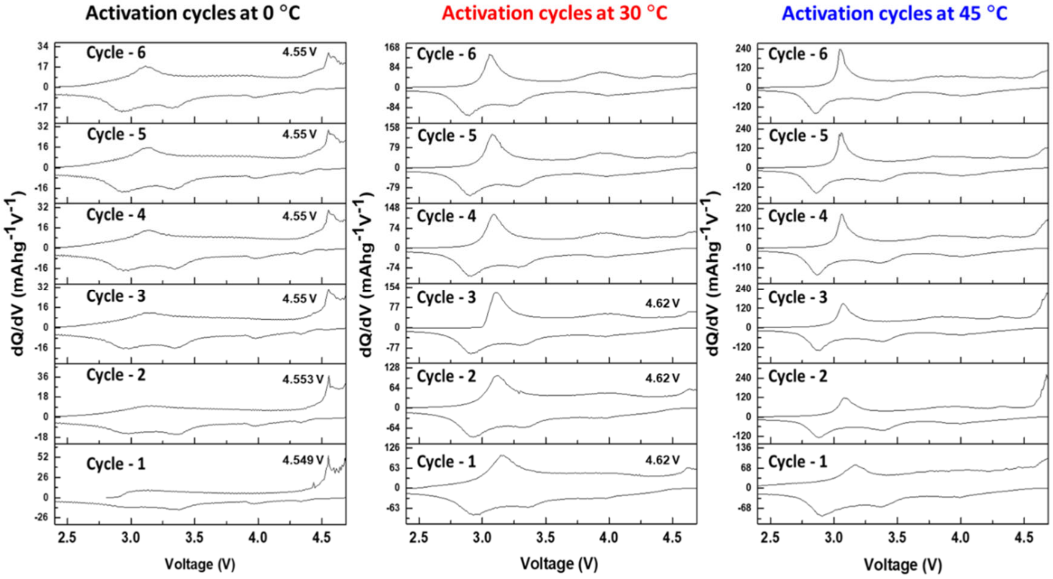

Figure S4. Differential capacity dQ/dV profiles of Li_2MnO_3 electrodes measured during several initial cycles at 0 °C (**a**), 30 °C (**b**), and 45 °C (**c**) as shown in **Figure 3.**

Impedance Measurements and their Analysis by Impedance Spectroscopy Genetic Programing (ISGP)

To obtain precise information of different resistances and capacitances representing the electrochemical processes in Li_2MnO_3/Li cells, we have analyzed the data via ISGP that computes distribution function of relaxation times (DFRT) (Γ vs. log f). In general, for cathodes in Li-ion cells, a single arc at high frequencies corresponds to the surface film resistance (R_{sf}) and capacitance (C_{SEI}) from different layers of the solid electrolyte interphase formed on the active mass. In the mid frequency range the arc corresponds to the charge transfer resistance (R_{ct}) and the double layer capacitance (C_{DL}) and the spike at the low frequencies is due to the solid-state lithium-ion diffusion. Since the arcs are not semicircles, they are translated into wider peaks, rather than delta functions, in the DFRT, and we should talk about effective resistances and effective capacitances, which can be obtained directly from those peaks as it is described below. We have obtained three peaks from the whole spectrum conferring R_{sf}, C_{SEI}, R_{ct} , C_{DL} and the solid-state lithium ion diffusion respectively from different iterations in ISGP. Presently, the data has been used for ISGP fitting down to f = 1 Hz starting from f = 100 kHz. In this bandwidth, the EIS data does not comply with Kramers-Kronig transformations and therefore only the higher frequency peaks can be analyzed safely to provide peaks that are translated into effective resistance and capacitance. The lower frequency part provides a partially out-of-range peak in the DFRT.

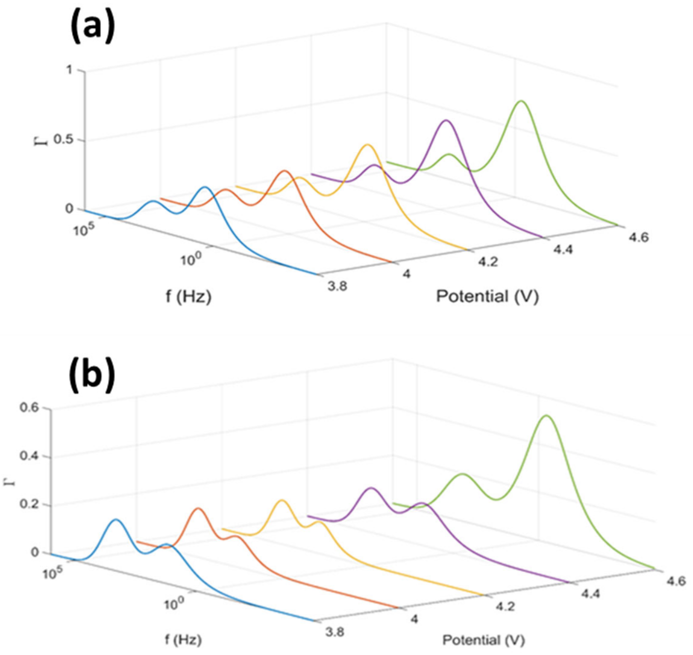

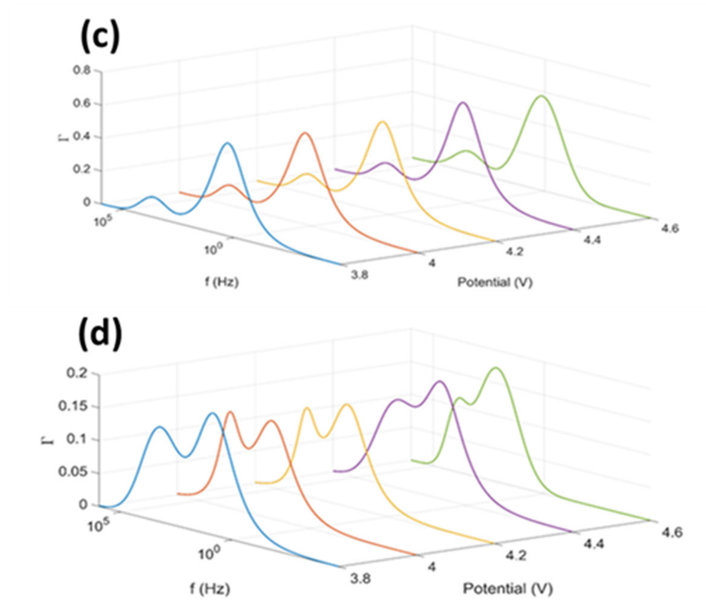

Figure S5. The DFRT for impedance of Li_2MnO_3 electrodes measured at 30 °C after initial activation cycles performed at 0 °C (a) and after subsequent cycling at 30 °C (b), and for similar Li_2MnO_3 electrodes measured at 30 °C after initial activation cycles performed at 30 °C (**c**) and after subsequent cycling at the same temperature of 30 °C (**d**) Peaks correspond to surface film (sf) and charge-transfer (ct) resistances. Typical impedance spectra of Li_2MnO_3 electrodes measured at 30 °C at 4.4 V upon charging are shown in **Figure S6.**

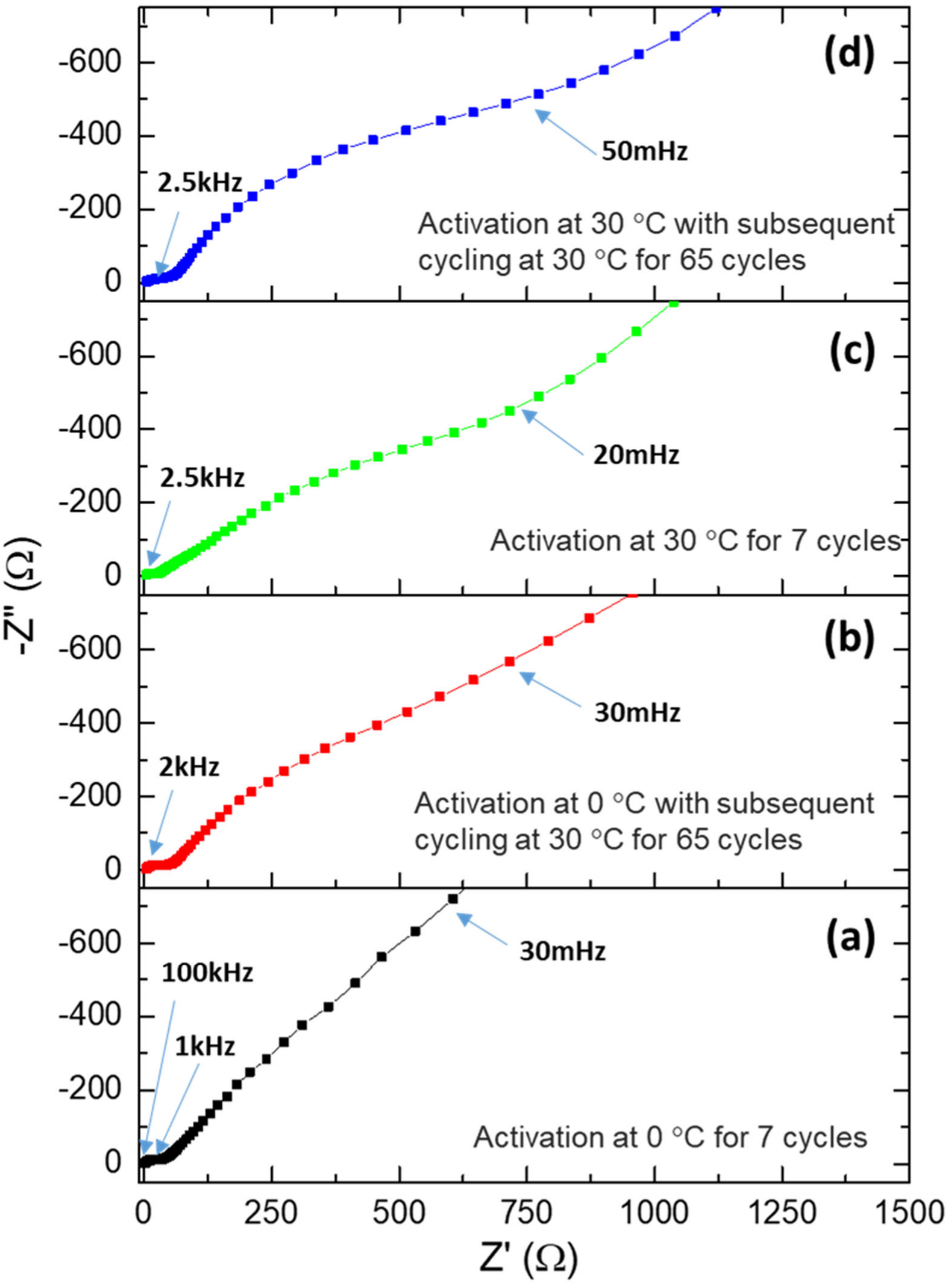

Figure S6. Typical impedance spectra (Nyquist plots) of Li_2MnO_3 electrodes measured at 4.4 V upon charging, at 30 °C. Four points of impedance measurements are marked with green stars in **Figure 3b**, namely: (**a**) after activation cycles at 0 °C; (**b**) after activation cycles at 0 °C with subsequent cycling at 30 °C; (**c**) after activation cycles at 30 °C; (**d**) after activation cycles at 30 °C with subsequent cycling at 30 °C.

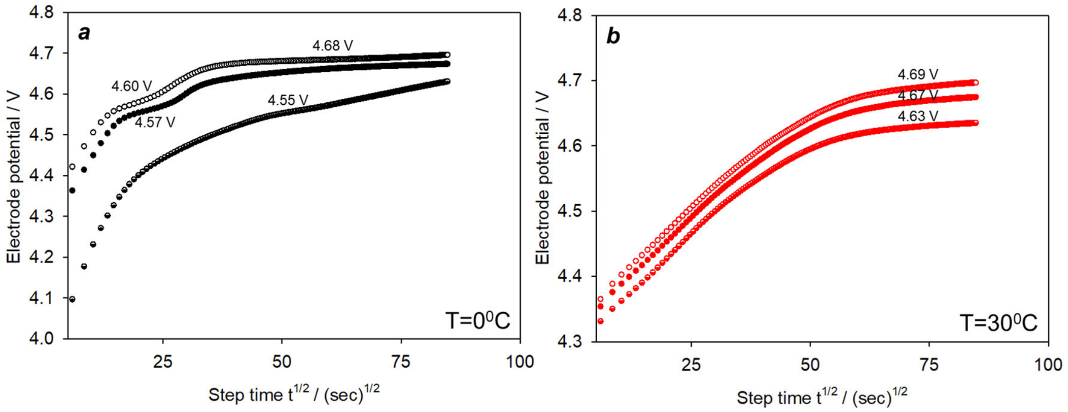

Figure S7. Variations with time of the transient electrode potential measured during the 1st charging process by three subsequent GITT titration stages of Li_2MnO_3 samples at 0 °C (**a**) and 30 °C (**b**). Coin-type cells, LP-57 solution. The current density applied was ~4 μA/cm² corresponding to ~2 mA/g. Semi-filled, filled, and empty symbols relate to OCV potentials of 4.10 V, 4.36 V, and 4.42 V in (**a**) and 4.33 V, 4.35 V, and 4.36 V in (**b**), respectively.

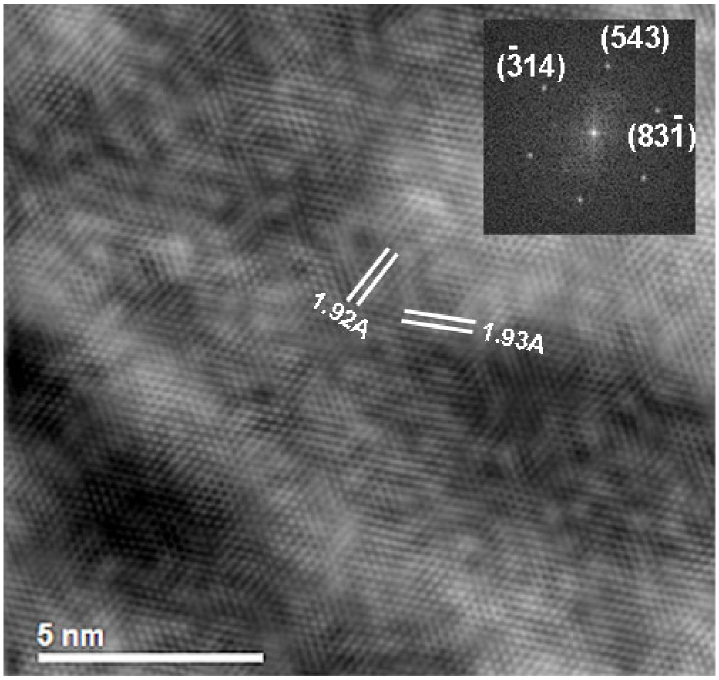

Figure S8. High-resolution image taken from the particle shown in **Figure 7a.** This particle was identified as the tetragonal spinel $Li_2Mn_2O_4$. The indexed Fourier transform shown in the insert is in accordance with the corresponding CBED pattern in **Figure 7a.**

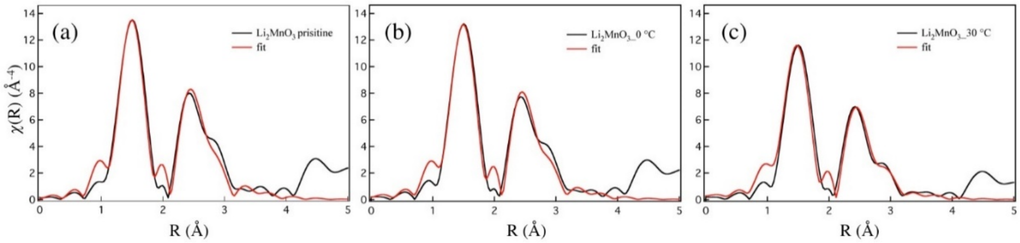

Figure S9. Mn K-edge Fourier transform magnitudes of k^3-weighted EXAFS data and theoretical fits of Li_2MnO_3 materials: pristine powder (**a**) and electrodes after the 1st cycle at 0 °C (**b**) and at 30 °C (**c**).

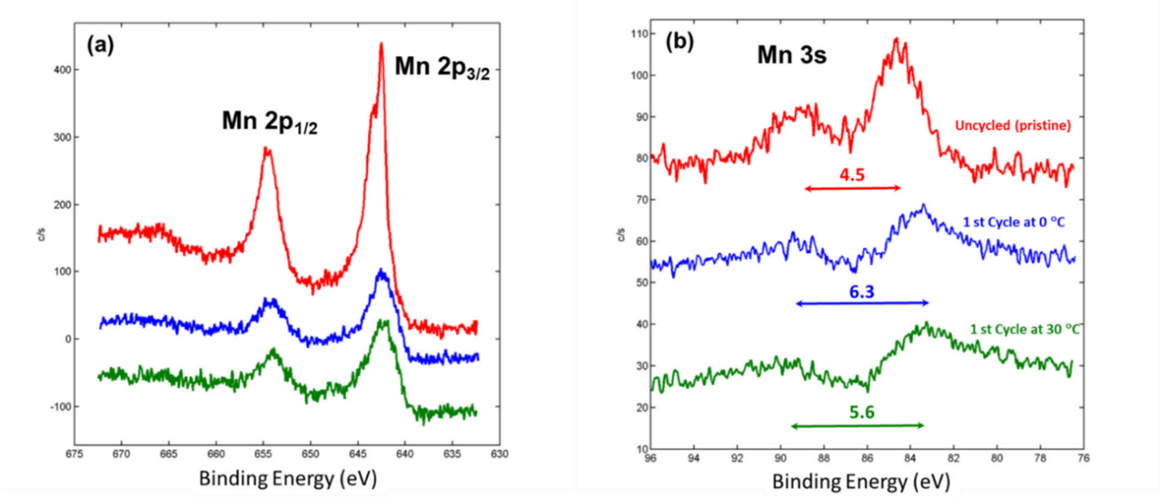

Figure S10. Mn 2p and Mn 3s XPS spectra, (**a**) and (**b**), respectively measured from uncycled (pristine) Li_2MnO_3 electrode (red lines) and those subjected to the first charge-discharge cycles at 0 °C and 30 °C (green and blue lines, respectively). The Mn 3s spectra include color-coded values for the magnitude of the splitting Δ, eV between the two peaks. Voltage profiles of these Li_2MnO_3 electrodes are shown in **Figure 2a** and **b.**

Table S1. Cell parameters of Li_2MnO_3 materials calculated from the corresponding XRD patterns.

**Material**	**Cell parameters of the monoclinic phase Li_2MnO_3**	**Reliability factor (%)**
Powder (pristine)	a = 4.926 Å, b = 8.512 Å, c = 5.022 Å<br>β = 109.33°	R_p = 2.55
Electrode (pristine)	a = 4.932 Å, b = 8.489 Å, c = 5.004 Å<br>β = 109.10°	R_p = 7.97
Electrode after<br>first cycle at 0 °C (2 V)	A = 4.932 Å, b = 8.492 Å, c = 4.996 Å<br>β=109.22°	R_p = 7.42
Electrode after<br>first cycle at 30 °C (2 V)	a = 4.921 Å, b = 8.527 Å, c = 5.012 Å<br>β = 109.40°	R_p = 7.05

Table S2. Content (in %) of the lattice oxygen in Li_2MnO_3, per-oxo like and other components as calculated by fitting of the corresponding O 1s spectra measured from pristine (uncycled) Li_2MnO_3 electrode and those after initial activation cycles at 0 °C and 30 °C. The corresponding binding energies (in eV) are shown in blue color.

Li_2MnO_3 electrode	O in Li_2MnO_3	Per-oxo like species O_2^{2-}	LiOH/ C=O	Li_2CO_3	O-C	F-C-O
Pristine (uncycled)	529.85		531.26	532.23	533.18	534.39
	48.9%	-	10.6%	23.5%	11.9%	5.1%
Initial cycling at 0 °C	529.85	530.65	531.26	532.23	533.18	534.29
	5.3%	5.2%	13.0%	39.2%	25.9%	11.4%
Initial cycling at 30 °C	529.85	530.65	531.35	532.23	533.18	534.29
	6.0%	11.6%	22.8%	36.9%	16.8%	5.9%

Table S3. Best fit results for the structural parameters obtained by analysis of the Mn K-edge EXAFS data of Li_2MnO_3 samples: pristine powder and electrodes after the 1st cycle (charge to 4.7 V, discharge to 2.0 V) at 0 °C and 30 °C. N: coordination number; R: interatomic distance; σ^2: Debye-Waller factor (mean-square disorder in R); ΔE_o: Energy shift; S_0^2 (amp): the amplitude reduction factor.

Sample	Bond	N	R (Å)	σ^2 (Å^2)	ΔE_o (eV)	S_0^2
Pristine powder	Mn-O_1	6	1.90 ± 0.01	0.001 ± 0.002	4.8 ± 1.5	0.58 ± 0.08
	Mn-Mn	3	2.86 ± 0.01	0.002 ± 0.001	4.8 ± 1.5	0.58
	Mn-O_2	8	3.63 ± 0.08	0.015 ± 0.014	4.8 ± 1.5	0.58
Electrode after the 1st cycle at 0 °C	Mn-O_1	6	1.90 ± 0.02	0.002 ± 0.001	4.7 ± 2.2	0.58
	Mn-Mn	3	2.86 ± 0.02	0.002 ± 0.002	4.7 ± 2.2	0.58
	Mn-O_2	8	3.61 ± 0.11	0.014 ± 0.018	4.7 ± 2.2	0.58
Electrode after the 1st cycle at 30 °C	Mn-O1	6	1.90 ± 0.01	0.003 ± 0.001	3.1 ± 1.3	0.58
	Mn-Mn	3	2.87 ± 0.01	0.003 ± 0.001	3.1 ± 1.3	0.58
	Mn-O_2	8	3.58 ± 0.06	0.014 ± 0.009	3.1 ± 1.3	0.58
